# Exploration of the potential mechanism of Baicalin for hepatic fibrosis based on network pharmacology, gut microbiota, and experimental validation

**DOI:** 10.3389/fmicb.2022.1051100

**Published:** 2023-01-04

**Authors:** Sujie Liu, Pingping Chen, Shadi A. D. Mohammed, Zihui Li, Xin Jiang, Juan Wu, Shumin Liu

**Affiliations:** ^1^Graduate School of Heilongjiang University of Chinese Medicine, Harbin, Heilongjiang, China; ^2^Institute of Traditional Chinese Medicine, Heilongjiang University of Chinese Medicine, Harbin, Heilongjiang, China; ^3^School of Pharmacy, Lebanese International University, Sana’a, Yemen; ^4^College of Life and Health, Dalian University, Dalian, China

**Keywords:** Baicalin, hepatic fibrosis, network pharmacology, gut microbiota, gut-liver axis

## Abstract

Baicalin (BA) is among the most effective and abundant flavonoids extracted from Scutellaria baicalensis that may be utilized to treat diseases associated with hepatic fibrosis (HF). Through network pharmacology, gut microbiota, and experimental validation, this research intends to elucidate the multi-target mechanism of BA on HF. BA targets were screened using databases and literature. As a result, In the anti-HF mechanism, the BA and 191 HF-associated targets interact, with 9 specific targets indicating that the BA’s anti-HF mechanism is closely linked to gut microbiota. Consequently, rat intestinal content samples were obtained and examined using 16S rRNA sequencing. In the BA-treated group, the gut microbiota was positively regulated at the phylum,and genus levels, with Lactobacillus performing significantly. The study concluded that BA has a multi-targeted anti-HF effect and has changed the gut microbial ecosystem.

## Introduction

Hepatic fibrosis (HF) is a reversible dynamic process caused by an excess of extracellular matrix (ECM) proteins that generate fibrous scars consisting of type I and type III collagen (Friedman, 2003; Kisseleva and Brenner, 2021). HF is classified into four stages of increasing severity, with stages 3 and 4 representing cirrhosis (Ginès et al., 2021). HF is the process that leads to the development of numerous chronic liver diseases, such as cirrhosis and hepatocellular cancer. According to statistics, 106 million individuals worldwide have decompensated cirrhosis, and 112 million have compensated cirrhosis. In 2017, it was reported that more than 1.3 million individuals worldwide died of liver cirrhosis, accounting for 2.4% of all mortality (Sepanlou et al., 2020). However, there is currently a shortage of western medications for the treatment of HF (Kisseleva and Brenner, 2021; Wang Z. Y. et al., 2021). Increasing data suggest that natural chemicals derived from medicinal plants play an essential role in treating chronic liver diseases (Latief and Ahmad, 2018; Perumpail et al., 2018; Chen et al., 2022).

**Figure 1:**
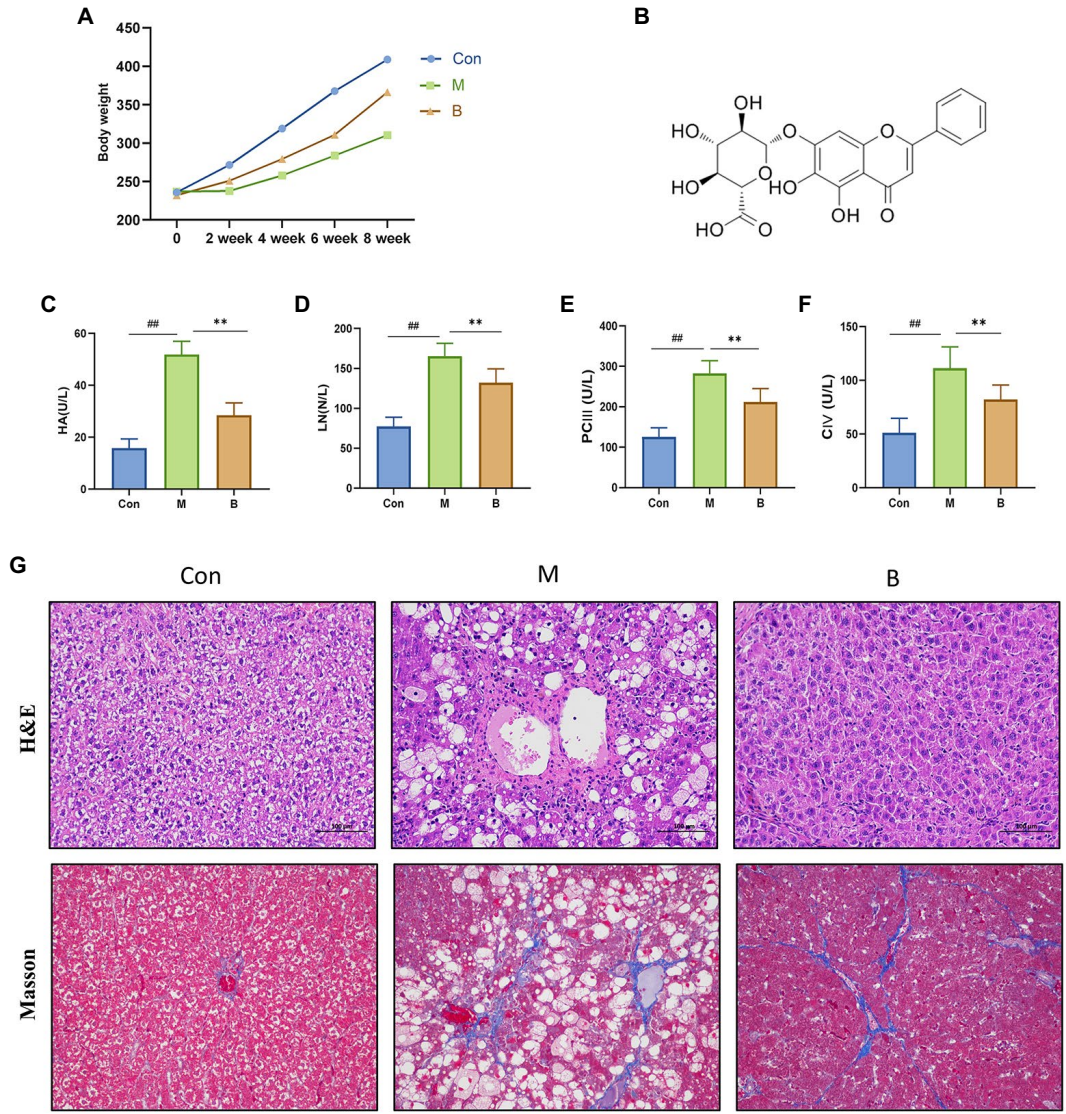
Establishment of HF model and curative effect of BA. **(A)** Body weight, **(B)** BA chemical structure, **(C)** Serum HA (U/L), **(D)** Serum LN (U/L), **(E)** Serum PC III (U/L), **(F)** Serum CIV (U/L), **(G)** Hematoxylin–eosin (H&E) staining and Masson staining (magnification, × 200). compared with the Con group, ^##^*p* < 0.05; ^##^*p* < 0.01; compared with the M group, *p < 0.05; **p < 0.01.

Baicalin (BA) is among the most potent and plentiful flavonoids extracted from Scutellaria baicalensis, and its chemical structure is shown in Figure 1B. Previous studies have shown that BA has antioxidant (Shi et al., 2018), anti-inflammatory (Zhang et al., 2022), anti-apoptosis (Zhao et al., 2022), antiviral (Zhao et al., 2018) and other effects. Recently, there has been an increasing focus on its ability to protect against liver and intestinal tract diseases (Liao et al., 2020). BA has effects on several liver disorders, including acute liver injury (Shi et al., 2020), fatty liver (Dai et al., 2018), non-alcoholic fatty liver disease (Junior et al., 2021), liver tumor (Jiang et al., 2022), and HF (Yang et al., 2012). Simultaneously, BA may control enteric-related disorders and protect the intestine against sepsis and ulcerative colitis (Zhu et al., 2020). Several trials have also proven BA to alleviate liver disease through the hepatointestinal axis (Zhang et al., 2022).

Network pharmacology, an emerging subject based on systems biology theory, is a network-based technique for researching complicated diseases with therapeutic targets and biomarkers (Barabási et al., 2011). The network pharmacology technique has been used to examine “compound-proteins/genes-disease” pathways, which are capable of expressing complexity between biological systems, medications, and diseases from a network viewpoint and share a holistic philosophy as TCM. The use of systems biology methodologies to identify the pharmacological activity, mechanism of action, and safety of TCMs is very useful for current TCM research and development (Zhang et al., 2019; Zhou et al., 2020). Furthermore, recent studies have shown that natural compounds may relieve HF by targeting gut microbiota and modifying the gut microbial ecosystem, and these results demonstrate that natural compounds can alleviate HF by targeting gut microbiota and altering the gut microbial ecosystem. By modulating intestinal flora, targeted supplementation may ameliorate HF and intestinal injury in rats (Lee G. et al., 2020). There are study baicalin attenuate diet-induced metabolic syndrome by improving gut microbiota (Lin et al., 2022).

**Figure 2:**
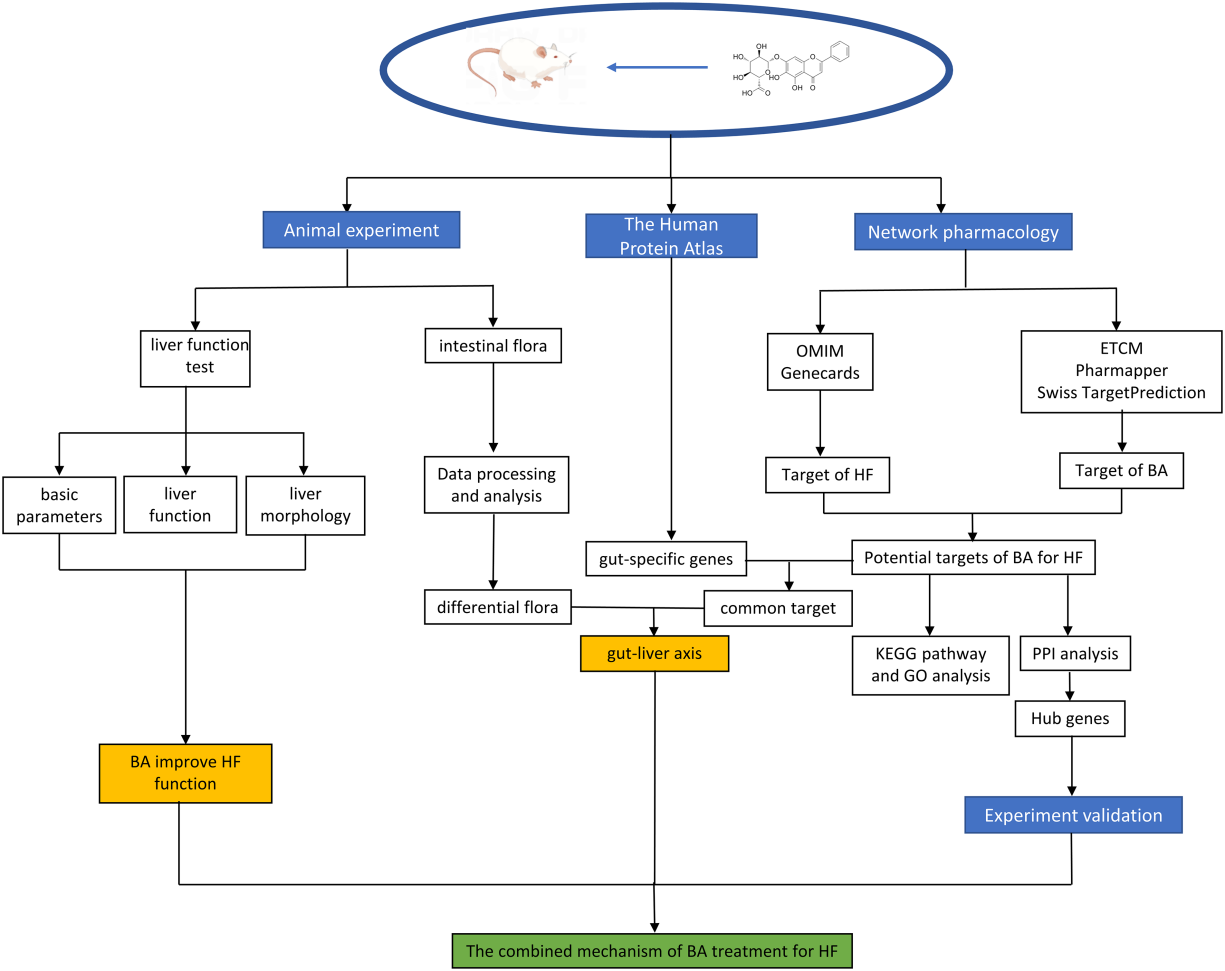
Flowchart of the experiment.

Previous research has indicated that BA can cure HF *via* various methods; however, further research is necessary to confirm how BA and anti-HF targets alleviate HF by boosting the quantity and activity of particular gut microbiota. In this research, by utilizing bioinformatics techniques. we combined network pharmacology with gut-specific expression analyses and 16S rRNA sequencing to explore and predict how natural compounds regulate HF through gut microbiota. The flowchart is shown in Figure 2.

## Materials and methods

### Animals and experimental protocols

The SPF grade SD male rats (220 ± 20) were provided by the Experimental Animal Center of Heilongjiang University of Traditional Chinese Medicine (certificate number: SYXK (Black) 2018–007). Feeding conditions were as follows: room temperature (21 ± 3)°C, relative humidity of 45–65%, adequate ventilation, rotating light and dark light for 12 h. Rats were allowed water and laboratory food *ad libitum*. This experiment was approved by the ethical committee of Heilongjiang university of Chinese medicine (Approved No DXLL2020081601). After 1 week of adaptation, the rats were randomly assigned to one of three groups: control group (Con), model group (M), and baicalin group (B). The M and B groups received intraperitoneal injections of CCl4 (40% in olive oil) at a dose of 3 ml kg-1 (5 ml kg-1 for the initial injection), whereas the Con group was injected with the same quantity of normal saline. All groups received their dosages twice a week for 8 weeks. BA administration started 4 weeks after modeling. According to the previous pharmacodynamic research and Chinese Pharmacopoeia, the dosage of BA was found to be 25 mg kg-1, which was supplied through gavage once per day for 4 weeks. After 8 weeks, all rats were anesthetized with 3% sodium pentobarbital solution and killed. Serum samples were collected from the abdominal aorta, centrifuged, and kept at −80°C to identify biochemical markers. The liver tissue was carefully separated, the middle lobe was preserved in 4% paraformaldehyde solution, and the cecum contents were rapidly transferred to a cryopreservation tube. All samples were frozen at −80°C until use.

### Pathological analysis

The liver tissue was taken from the 4% paraformaldehyde solution, fixated, dehydrated, transparent, wax dipped, and embedded into sections before being stained with hematoxylin and eosin (H&E) and Masson’s solution. The images were then examined under a light microscope at 200X magnification.

### ELISA detection

The Enzyme-linked immunosorbent assay (ELISA) was used to determine serum hyaluronic acid (HA, batch number: H021-2-2); laminin (LN, batch number: H012-2-3); type III procollagen (PCIII, batch number: H003-1-1); type IV collagen (CIV, batch number: H015-1-2) levels, using a microplate reader (Methermo company) for analysis.

### Network pharmacology analysis

#### BA structure information and ADME standards

The BA’s structure was obtained from the NCBI PubChem database^1^ (Wang et al., 2009), a public database of small molecules’ biological characteristics and chemical structures. Then, we evaluate BA using screening criteria in traditional Chinese medicine (TCMSP) database system pharmacology of absorption, distribution, metabolism, and excretion (ADME). There were two measures used: oral bioavailability (OB) and drug-likeness (DL) (Lee et al., 2018). OB indicates the proportion of an orally administered dosage of unaltered medication that enters the systemic circulation and becomes accessible at the site of action (Xu et al., 2012). DL can be used to describe molecular properties that have an impact on pharmacodynamics and pharmacokinetics (Tao et al., 2013). The OB 30% and DL 0.18 thresholds were used for both measurements (Yue et al., 2017; Wang et al., 2020).

#### Target screening of BA in the treatment of HF

The Pharmapper,^2^ ETCM^3^ databases, as well as the Swiss Target Prediction^4^ were utilized to identify probable BA target genes. After merging the database mentioned above targets, BA-related targets from the Uniport^5^ database are uniformly modified. The keywords “liver fibrosis” and “hepatic fibrosis” were used to search in the OMIM^6^ and Genecards^7^ databases for HF-related targets. Then, import the BA and HF disease target data into R (3.6.3) to determine the drug-disease target intersection and generate the VEEN map.

#### Construction of protein interaction network

First, input the targets obtained into the STRING^8^ database, then selected “Homo sapiens (Human)” as the species, then conduct protein–protein interaction (PPI) analysis. The points and overall scores were entered into Cytoscape 3.8.0 to create a protein interaction network. The node size indicates the degree of the node, with the greater the node size reflecting the higher the degree value. Simultaneously, the node color is proportional to the level of interaction.

#### Analysis of tissue-specific gene expression

The 941 enhanced genes were obtained from the Human Protein Atlas^9^ (on January 1, 2022) to identify tissue-specific targets in the colon and small intestine, which exhibited higher expression in the gut compared to other tissues. The combination of these two gene sets led to the identification of a BA gut-specific anti-HF target. Immunohistochemical antibody-based maps were used to gather protein expression data from the gut.

#### Functional enrichment analysis

The annotation, visualization, and comprehensive discovery database (DAVID) is utilized for enrichment analysis for KEGG and Gene ontology (GO) enrichment analysis of the biological process (BP), molecular function (MF), and cellular component (CC).

### Western blot analysis

The colon tissue total protein was isolated and measured using a BCA kit (Biotechnology). Proteins were separated using a 10% SDS-PAGE gel, and the PVDF membrane was blocked with 5% skim milk powder. The primary anti-phosphatidylinositol 3-kinase (AB22048, Abcam, AB182651, PI3K), protein kinase B (Abcam, AB81283, AKT), and target of rapamycin (mTOR) (Ptgcn,67,778-1-Ig, mTOR) antibodies were added, and HRP-conjugated secondary antibodies were added overnight. Image Pro Plus 6.0 was used to examine each band’s gray value. The relative protein expression level was determined by the ratio of the target protein band to β-actin.

### Immunohistochemical analysis

The immunohistochemistry detection was conducted strictly with the immunohistochemical kit’s instructions. After embedding, liver and colon tissues were sectioned, deparaffinized, hydrated, and cleaned. Sections were then treated with a pH 6.0 sodium citrate buffer before being blocked with 3% H2O2 and goat serum. The target protein was next stained with membrane protein (66452-1-Ig, proteintech, ZO-1) and transmembrane protein (ab211737, Abcam, Claudin-1). Observing and photograph DAB was darkened and developed under a microscope (motic, DMB5-2231P1), and brown was positive staining. Image Pro Plus 6.0 software was utilized for processing, and the integrated absorbance IA/area was employed as the semi-quantitative outcome of the detection index.

### Gut microbiota analysis

The intestinal contents of five rats were collected, and the Genomic DNA Kit (Omega Bio-tek, Inc., Norcross, GA, United States) was used to extract DNA from the rats’ feces, as previously mentioned (Fulle et al., 2000; Liu et al., 2021). The Primers were then used to amplify the 16S rRNA gene (V3-V4 region) from the whole genome (F,5-ACTCCTACGGGAGGCAGCA-3R,5-GGACTACHVGGGTWTCTAAT-3). After fluorescence quantification, all amplicons were purified, recovered, and sequenced on the Illumina MiSeq platform.

### Data analysis

GraphPad Prism 7 was utilized for analysis. The results are expressed as mean ± standard deviation. Comparisons between three groups were conducted using one-way ANOVA with Dennett’s multiple comparison test*p < 0.05, **p < 0.01 was statistically significant between groups. R software version 4.1.0 was used to conduct microbiological and structural equation modeling analyses. To create the rendered heatmaps, use the ggpubr package for principal component analysis, Cluster for hierarchical clustering, and the R heatmap package.

## Results

### Establishment of HF model and curative effect of BA

We assessed body weight once every 2 weeks for 8 weeks to determine how BA affected the physiological alterations in CCL4-induced HF mice. The body weight of rats in the M group was considerably different from that of rats in group Con after 4 weeks of modeling. The body weight of rats in group B increased significantly after treatment, as indicated in Figure 2. The four items of liver fiber were alleviated after BA administration. When compared to the Con group, the levels of HA, LN, PCIII, and CIV in M group serum were significantly increased (*p* < 0.01), while the levels of HA, LN, PCIII, and CIV in B group serum were significantly lower than in M group (*p* < 0.01). HE staining revealed that the hepatocytes in the Con group were tightly packed, while foam cells and a small number of lymphocytes penetrated from the hepatocytes to the model group. There was hepatocyte spot necrosis, nuclear fragmentation or disintegration, and enhanced eosinophilicity of the cytoplasm surrounding veins and arteries in the M group; connective tissue hyperplasia was also observed locally. In the BA group, several hepatocytes in the liver tissue were enlarged, and the cytoplasm was loose and light-colored, revealing small granules. These findings suggested that a CCL4-induced HF model in rats has been successfully created.

### Network pharmacology analysis of BA

#### BA structural and ADME-related information

The TCMSP is used to collect BA structural information (Supplementary Figure S1) as well as ADME-related information. The OB and DL of BA were following the estimated cut-off values of 40.12% and 0.75, respectively. This suggests that BA has comparable oral bioavailability to other medicines.

#### Target prediction and PPI core network analysis

**Figure 3:**
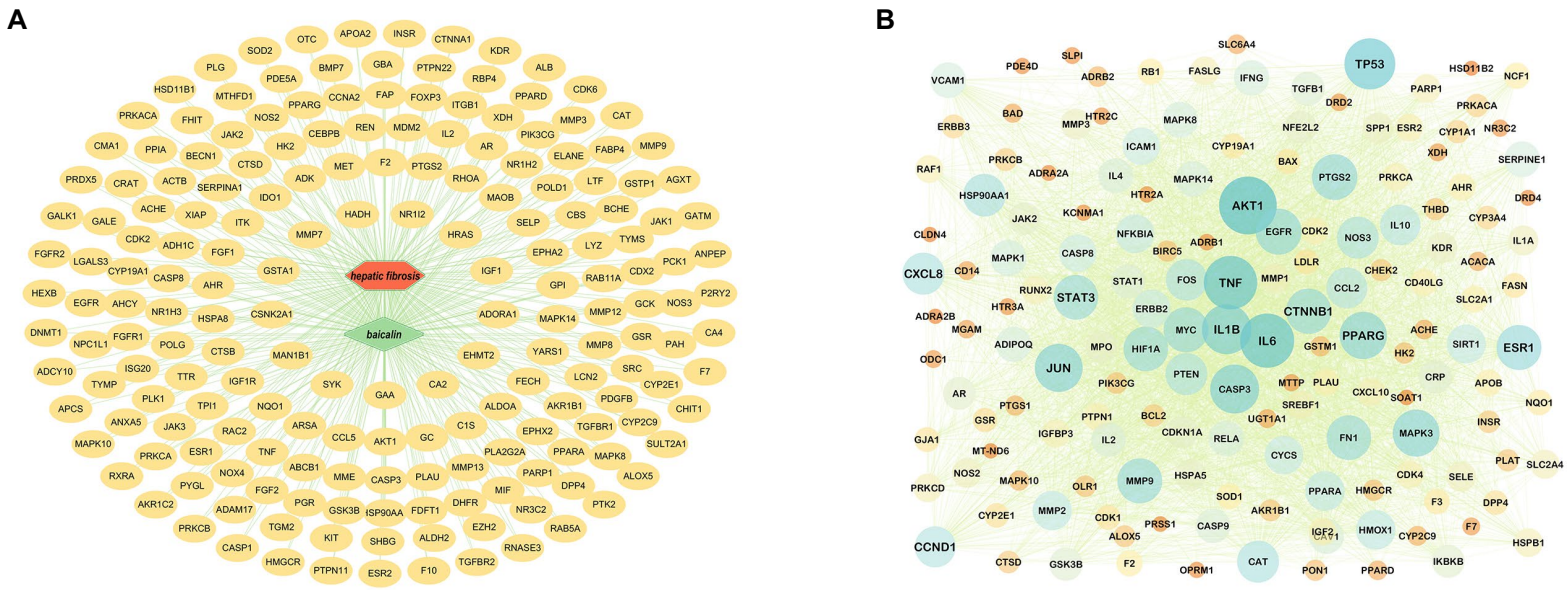
Target prediction and PPI core network analysis. **(A)** BA-targets (Green color in the center represents drug, red color represents diseases, and yellow color circles represent targets). **(B)**: PPI network diagram.

After removing the duplicated BA targets from the pharmMapper, SwissTargetPrediction, STITCH, and ETCM databases, a total of 442 non-repeated human protein targets of BA were found. Furthermore, the putative disease genes were screened using the scores supplied by the OMIM and GeneCards databases, after which the targets from these two databases were merged, duplicate values were removed, and eventually identified 1813 HF-related targets. Data on protein interactions were collected by entering 191 BA anti-HF targets into the String database. Using Cytoscape 3.8.0 software, these targets were mapped to the human protein–protein interaction network, giving a total of 156 targets and 3,539 edges (Figures 3A,B).

### Tissue target expression

**Figure 4:**
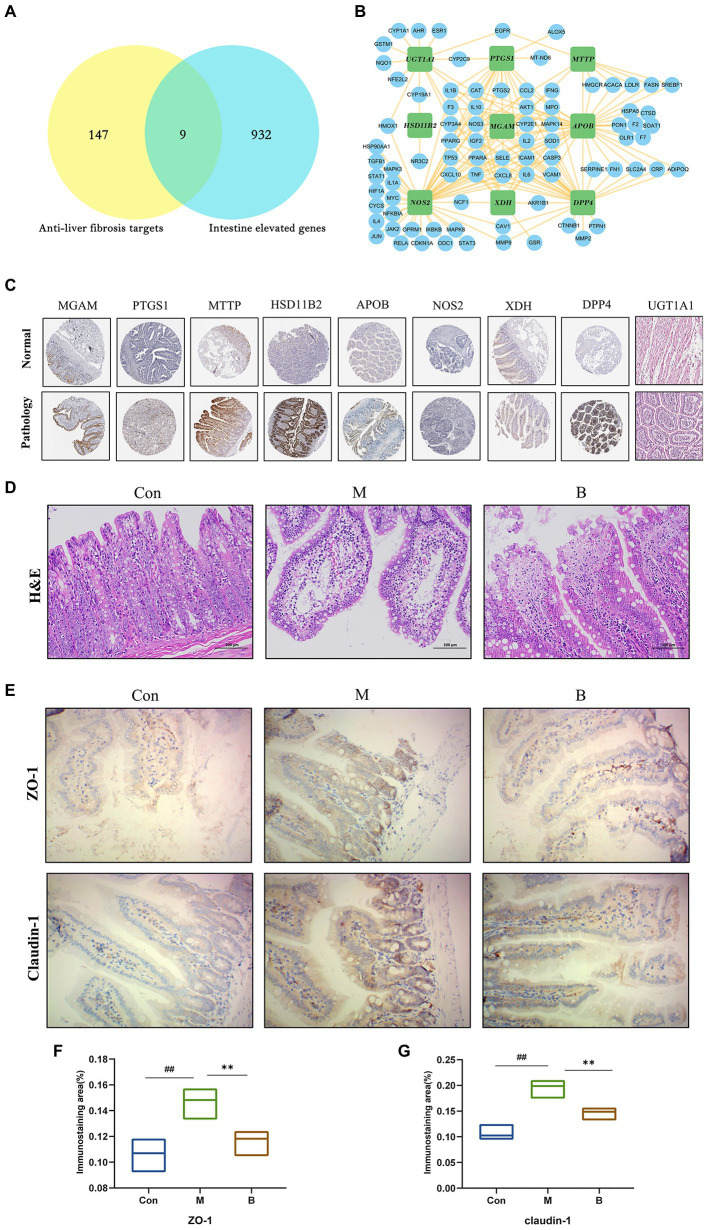
Tissue target expression **(A)** Venn diagram of gut-expressed genes and anti-HF targets. **(B)** Sub-network of nine targets: PTGS1, XDH, NOS2, DPP, MGAM, MTTP, APO, UGT1A, HSD11B2. **(C)** On normal and pathological tissues Immunohistochemical staining of nine intestinal abnormally expressed targets. **(D)** HE staining of the intestinal wall. **(E–G)** Immunohistochemical staining of intestinal wall permeability; compared with the Con group, ^#^*p* < 0.05; ^##^*p* < 0.01; compared with the M group, *p < 0.05; **p < 0.01.

The Human Protein Atlas was used to determine the specificity of gut-expressed targets. Venn diagrams were created using 191 anti-HF targets and 941 gut-elevated BA genes, yielding a total of 9 preferentially expressed targets in the gut. The data reveal that BA interacts with several possible targets in the gut in the management of HF. The intestinal-specific expressions of anti-HF were PTGS1 (colon), XDH (small intestine), NOS2 (colon), DPP4 (small intestine), MGAM (colon), MTTP (small intestine), APOB (small intestine), UGT1A1 (small intestine), and HSD11B2 (small intestine). A sub-network comprising nine gut-specific targets was identified. Most components are interconnected, suggesting that they perform comparable biological activities. We obtained immunohistochemistry staining of nine gut-specific expression targets from the Human Protein Atlas database to further examine, demonstrating that these proteins are differentially expressed across diseased tissue and normal colon samples. Simultaneously, changes in intestinal morphology suggest a possible involvement in intestinal function, such as the intestinal barrier system, intestinal metabolism, or permeability (Figures 4A,C,D).

The typical tight junction proteins claudin-1 and ZO-1 substantially impact the tight junctions and permeability of the intestinal epithelium. The structure of each layer of intestinal tissue in the Con group is obvious; the mucosal epithelium is intact, cell morphology is normal, and no visible inflammatory alterations are identified; in the M group, there is edema in the intestinal mucosal layer and dilated central chyle duct. The B group’s intestinal villi were shorter than those in the K group, and there was only a little degree of lymphocyte infiltration in the intestinal tissue. In terms of mRNA and protein levels, the expression levels of claudin-1 and ZO-1 in the M group were significantly lower than the Con group. In contrast, claudin-1 and ZO-1 expression levels in the B group were significantly higher than in the M group. Taken together, BA’s anti-HF mechanism is thought to be strongly related to its crucial function in gut microbes and host health (Figures 4E–G).

### Improvement of bacterial diversity and richness of rat gut microbiota

**Figure 5:**
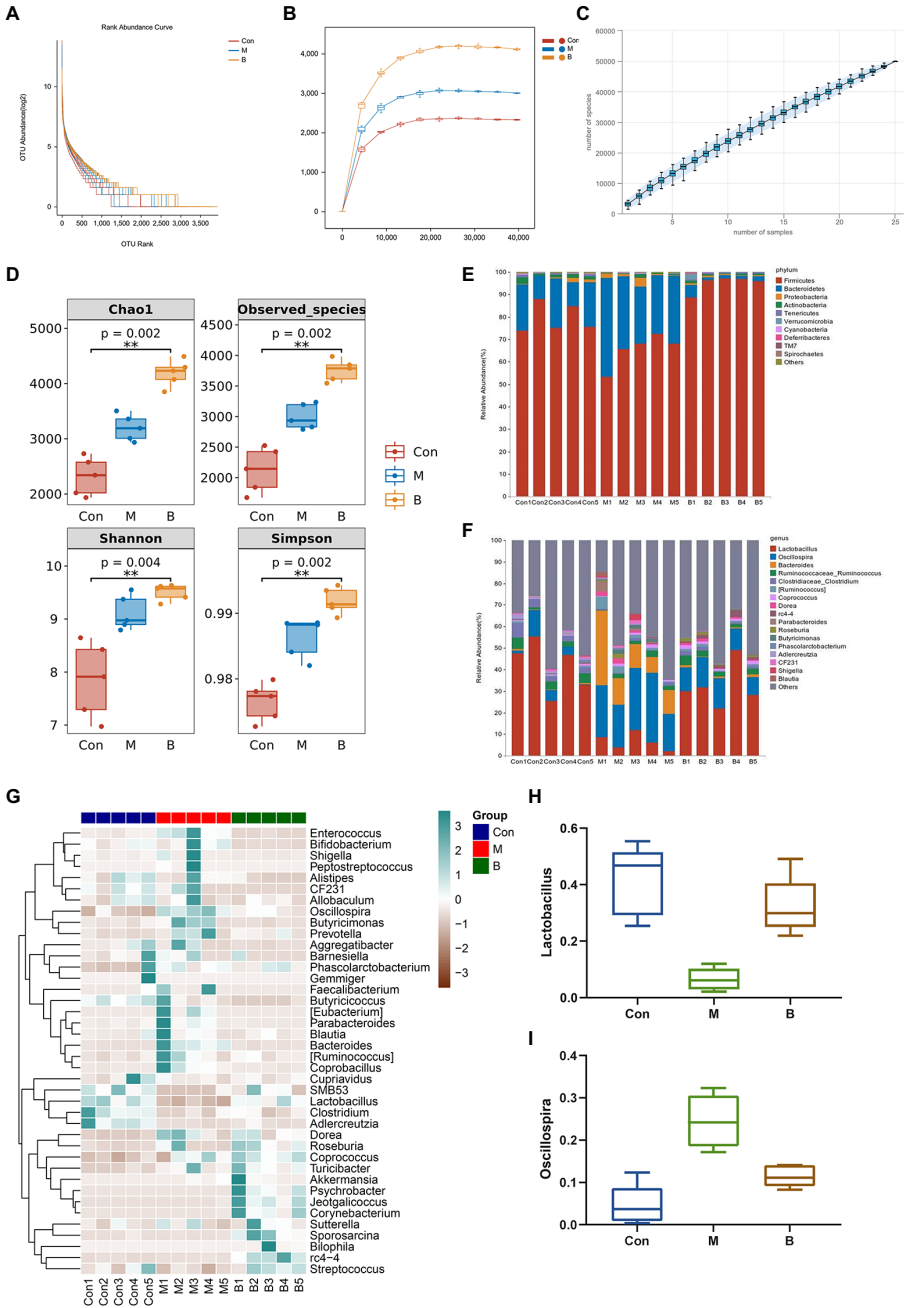
Gut microbiota alteration and variations **(A)** Rank abundance curve, **(B)** Sparse Curve, **(C)** Species accumulation curve, **(D)** alpha diversity, **(E)** Phylum level, **(F)** Genus level, **(G)** Heatmap, **(H)** Lactobacillus (relative abundance), **(I)** Oscillospira (relative abundance).

Many studies have revealed that gut microbiota homeostasis is important in preserving the gut epithelial barrier’s function and enhancing the intestinal epithelium’s immune protection (Granado-Serrano et al., 2019). Most gut bacteria can be annotated to the genus level, and the ranking abundance curves may show the number of species in the gut microbiota. As shown in Figure 5A, the rank abundance curve drops gradually and terminates at that point. Flattening suggests that the species sample is numerous and uniform. The sparseness curve in Figure 5B is essentially steady, indicating a reasonable sequencing depth. Furthermore, we examined alpha diversity across all samples to determine if BA influences diversity. The species accumulation curve demonstrates that the curve has slowly increased, indicating that the sample size is sufficient to represent the community’s species composition (Figure 5C). Chao1 and observed species indices may reveal species richness, while Shannon and Simpson indices, which represent gut microbial diversity, validate microbial diversity (Figure 5D). As seen in Figure 5E, the relative abundances of Firmicutes and Bacteroidetes in the phyla altered dramatically. In group M, the relative abundance of Bacteroidetes grew while the relative abundance of Firmicutes declined, while in group B, the relative abundance of Firmicutes increased while Bacteroidetes decreased. The relative abundance of s decreased, showing that BA greatly enhanced Firmicutes while suppressing Bacteroidetes. Correlations between important OUTs were illustrated by a heat map to evaluate microbial community alterations at OUT levels following BA use (Figure 5G). The samples’ genera were studied to learn more about the particular genera that metabolize BA (Figures 5F,H,I). As a result of BA intervention, the relative abundance of Lactobacillus increased,whereas the relative abundance of Oscillospira decreased significantly. Recent research has demonstrated that Lactobacillus, as an ingested microbial probiotic, may effectively prevent oxidative liver damage (Saeedi et al., 2020), alleviate NAFLD by modifying gut microbiota and inflammatory pathways (Lee N. Y. et al., 2020), and plays a role in the intestine and liver homeostasis (Santos et al., 2020). Oscillospira, an anaerobic bacterium, may delay the distribution of gut microbiota in NASH progression (Juárez-Fernández et al., 2022). These data suggest that the gut microbiota may play a role in modifying BA’s anti-HF effects. This indicates that BA, as a natural compound, alters the gut microbiota and reshapes the gut microbial ecosystem effectively.

### Go and KEGG enrichment analysis

**Figure 6:**
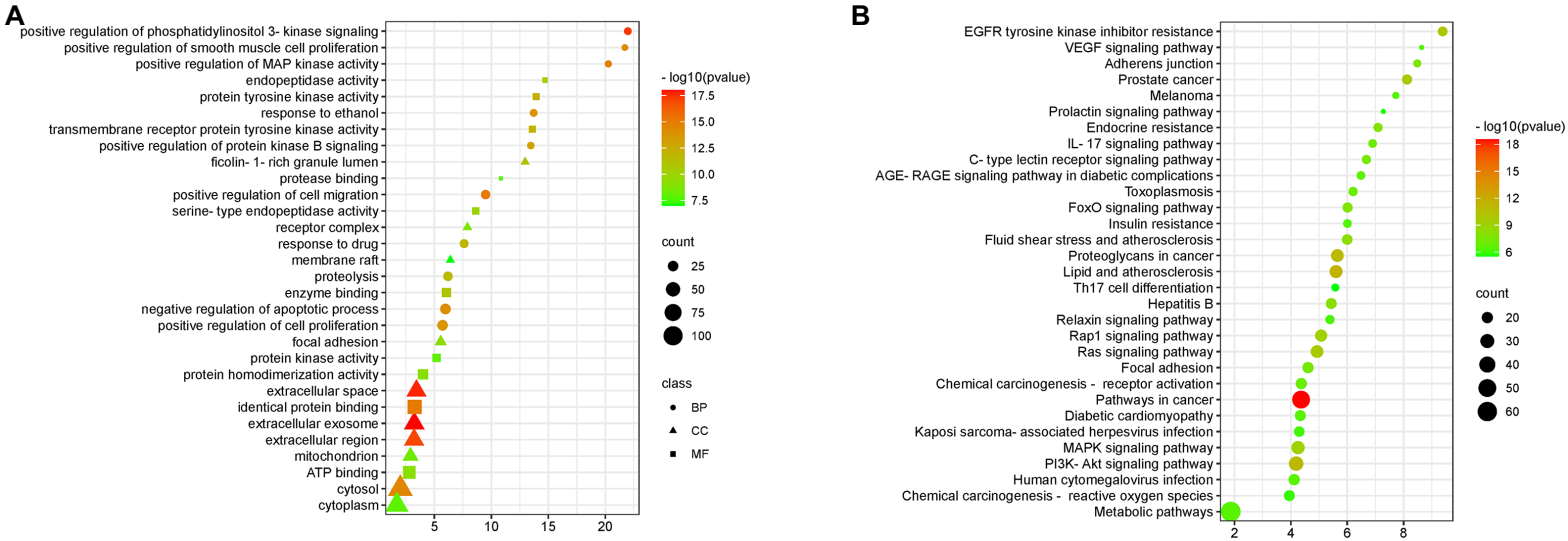
GO and KEGG enrichment **(A)** BA enrichment analysis of HF, **(B)** Enrichment analysis of signaling pathways affected by BA on HF.

Gene enrichment analysis was used to investigate the biological processes and signal pathways of 191 key targets to understand BA’s potential anti-HF effect. The findings revealed that the biological process was mainly involved in controlling apoptosis and cell proliferation, and the signal pathway was primarily involved in the IL-17 signal pathway, and VEGF signal pathway, as shown Figure 6.

### Experimental verification

**Figure 7:**
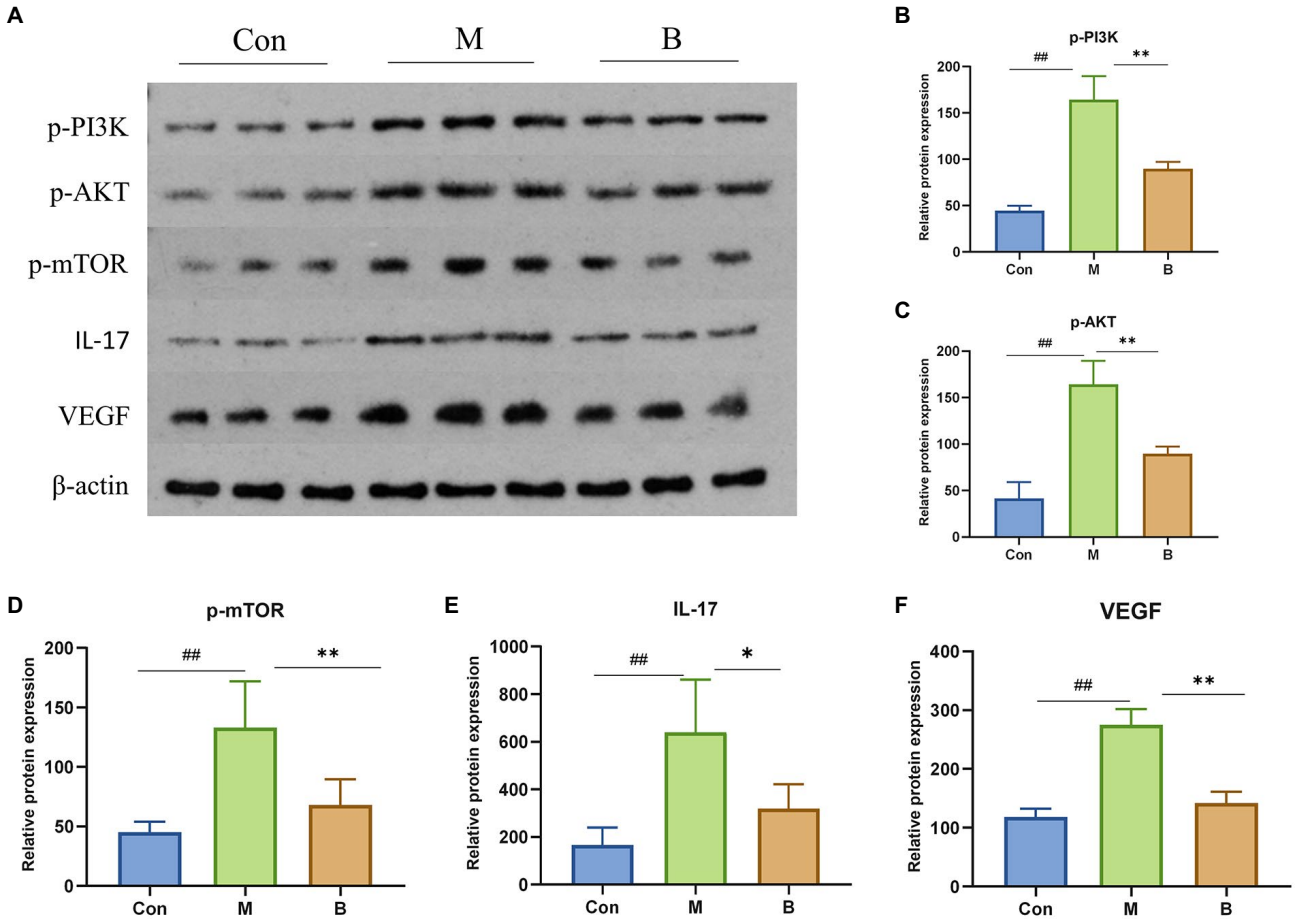
Protein expressions of PI3K, AKT, mTOR, IL-17 and VEGF in liver **(A)** PI3K, AKT, mTOR, IL-17, VEGF protein expression in liver tissue **(B–F)** *n* = 3, ^##^*p* < 0.01; compared with the Con group, ^#^*p* < 0.05; ^##^*p* < 0.01; compared with the M group, *p < 0.05; **p < 0.01.

Western Blot verified the mechanism of BA inhibiting HF. In contrast to the Con group, M group significantly enhanced the phosphorylation of PI3K (*p* < 0.01), AKT (*p* < 0.01), mTOR (*p* < 0.01), and the expression of IL-17 and VEGF, while group B significantly decreased the phosphorylation of PI3K (*p* < 0.01), AKT (*p* < 0.01), mTOR (*p* < 0.01), IL-17 (*p* < 0.05), VEGF (*p* < 0.01), IL-17 (*p* < 0.05), and VEGF (*p* < 0.01) levels compared to M group. It indicated that BA could directly inhibit PI3K/AkT, IL-17 and VEGF signaling pathways, as indicated in Figure 7.

## Discussion

**Figure 8:**
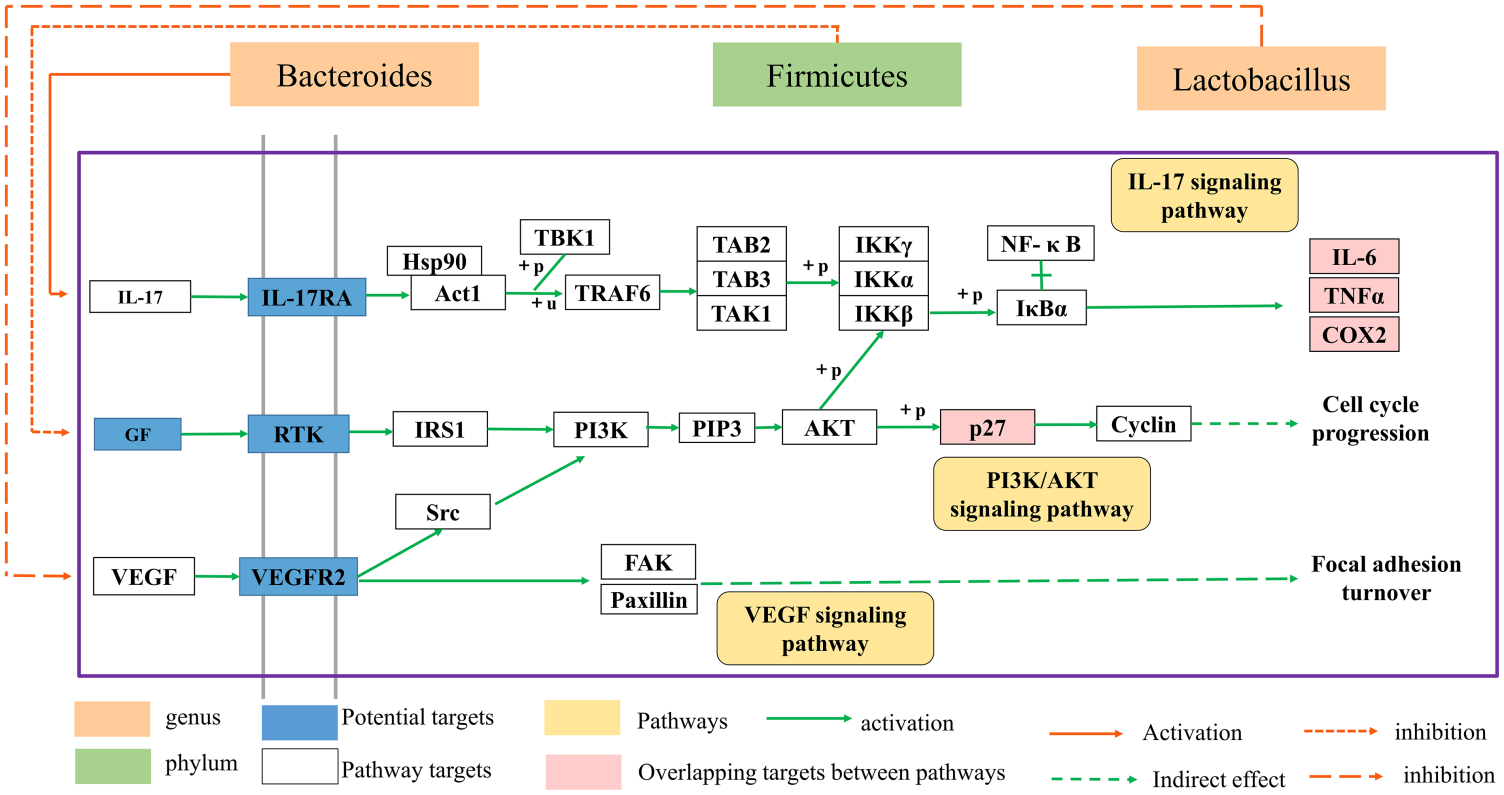
The relationship between the gut microbial ecosystem and the PI3K/AkT, IL-17, and VEGF signaling pathways.

HF is a transitional stage between chronic inflammation and cirrhosis, and the disease process is often thought to be reversible (Sun and Kisseleva, 2015). As a result, the therapy of HF has significant clinical importance as well as theoretical viability. In recent years, the extraction of high-efficiency, low-toxicity natural chemicals from medicinal plants for HF relief has attracted a lot of interest. Previous research has revealed that BA may have varying degrees of therapeutic impact on many liver diseases through oxidative stress, inflammation, lipid metabolism, and so on (Shen et al., 2017; Dai et al., 2018; Li et al., 2022). However, the mechanism of BA against HF remains unknown. Therefore, we constructed a rat model of HF using CCl4 to investigate the potential mechanism through which BA inhibits HF (see Figures 6–8).

CCl4-induced HF is a well-known animal model of chronic liver injury. This model’s symptoms are comparable to those of chronic liver damage in humans (Risal et al., 2012). An 8-week HF model produced by CCL4 was employed in this study, and histological and biochemical analyses of CCL4-injected rats were performed to confirm rat HF. Our findings revealed that BA decreased the area of collagen fibers in fibrotic liver tissue and increased the levels of HA, LN, PCIII, and CIV in the serum of rats in group M, while group B considerably improved the above alterations in fibrotic rats, showing that BA has an anti-HF effect.

This research employed a network pharmacology analysis approach to predict the anti-HF impact of BA and identified 191 potential targets of BA against HF in order to better identify the pharmacodynamic mechanism of BA in HF. Simultaneously, numerous studies have indicated that changes in the gut microbiota and intestinal permeability cause gastrointestinal diseases and lead to intestinal barrier disruption, abnormal bacterial debris and products, and other pathogen-related molecular patterns penetrate the liver through the portal circulation, causing the production of pro-inflammatory cytokines and thereby accelerating the onset of HF (Kumar et al., 2011; Zhou et al., 2019). Our nine enriched gut-specific targets are essential in controlling intestinal and hepatic diseases. For instance, NOS2 may reduce HF (Cinar et al., 2016; Shen et al., 2017; Becerril et al., 2019) and liver injury (Li D. et al., 2020) by reducing inflammatory responses, and studies has shown that NOS2 may reduce inflammatory bowel disease and maintain gut microbial homeostasis (Matziouridou et al., 2018). According to the findings of Gao et al. (2018), gut microbiota has a regulating influence on intestine UGT1A1. Xu et al. (2020) observed that the mRNA level of UGT1A1 was increased in chronic CCl-induced liver fibrosis rats (Xu et al., 2020). UGT1A1 may control bile acid metabolism, reducing cholestasis-induced liver injury (Wu et al., 2021), while APOB RNA expression has been reported to be decreased in hepatocellular carcinoma (Adrian et al., 2017). The experimental work conducted by Bo-Tao Li et al. revealed that an APOB stability deficiency is a major contributor to nonalcoholic fatty liver disease (Li B. T. et al., 2020). Kyung Eun Yun’s research found that APOB was substantially linked with the microbial diversity of the gut microbiota (Yun et al., 2020).

Meanwhile, in this experiment, we discovered that a high number of intestinal glandular epithelial cells degeneration, cell swelling, villus disintegration, and fragmentation occurred in the intestinal wall of rats treated with CCl 4, as previously established by Wan et al. (2019) and Yan et al. (2019) studies. The diseased state of the intestinal wall was relieved following BA therapy, and the intestinal villi were more uniform and tidy. More critically, tight junction proteins are indicators of intestinal barrier integrity and are crucial for maintaining intestinal integrity (Zeisel et al., 2019). As a result, we investigated at important intestinal barrier indicators such as ZO-1 and Claudin-1. CCL4 considerably reduced the expression of these genes, but BA dramatically increased the expression of ZO-1 and Claudin-1. BA was shown to be efficacious in preventing CCL4-induced intestinal epithelial barrier disruption. Furthermore, Wang C. et al. (2021) investigation yielded similar findings.

Numerous studies have revealed that disruption of the intestinal microbiota plays a crucial role in the development of HF (Yu and Schwabe, 2017). This study utilized 16S rRNA gene sequencing to investigate if the positive impact of BA is connected to the modulation of intestinal microbiota. Our findings demonstrated that BA has a strong regulating influence on gut microbiota. At the phylum level, the number of firmicutes in group B increased significantly compared to group M, whereas the number of bacteroides declined significantly. Our results reported similar to those of Wang C. et al. (2021). Furthermore, studies have indicated that increasing firmicutes might reduce the body’s inflammatory response (Samaddar et al., 2022). BA treatment enhanced the relative abundance of Lactobacilli while decreasing the relative abundance of Shivering spiralis at the genus level. Jantararussamee et al. revealed that lactic acid bacteria might preserve the liver by reducing fibrosis and inflammation in the injured liver (Jantararussamee et al., 2021). Overall, our findings suggest that BA may reduce HF *via* controlling gut microbiota, with the regulation mechanism most likely connected to inflammation.

The enriched signaling pathways were analyzed further to understand the underlying mechanism of BA on HF. Previous studies have shown that PI3K/AKT, IL-17 and VEGF signaling pathways are involved in the progression of HF (Wu et al., 2019; Yu et al., 2019). The deletion of Akt1 and Akt2 in the liver has been shown to cause liver inflammation and hepatocyte death, and the use of PI3K/AKT inhibitors has been shown to significantly enhance the degree of liver damage in mice (Reyes-Gordillo et al., 2019). Furthermore, the IL-17 and VEGF signaling pathways may contribute to a decrease in HF (Kumar et al., 2016; Fabre et al., 2018; Martínez-López et al., 2019; Qiao et al., 2020; Ghanim et al., 2021; He et al., 2022). Huiji Pan et al. discovered that gut microbiota influence H-L + HFD-induced liver fibrosis through alteration of the hepatic IRS1/Akt/mTOR signaling pathway (Pan et al., 2022). According to Jiang’s study (Jiang et al., 2020), Lactobacillus may enhance the intestinal milieu and alleviate the condition, and the level of VEGF is also dramatically lowered with the reduction of liver cancer. Wang et al. discovered that the differentiation of cytokines associated to the IL-17 signaling pathway is connected to intestinal flora colonization and may be involved in intestinal immunological homeostasis (Wang et al., 2019). Yingying Liu et al. discovered that HRG may enhance intestinal inflammation, immunology, and lipid production through similar pathways, therefore enhancing NAFL *via* modulating the intestinal flora (Liu et al., 2022). According to the findings of this research, BA may strongly suppress the production of p-PI3K, p-AKT, p-mTOR, IL-17, and VEGF, showing that BA can exhibit anti-HF actions through the PI3K/AkT, IL-17, and VEGF signaling pathways. As a result, we hypothesize that BA may ameliorate HF *via* modifying the gut microbiota and signaling pathways. However, our work has significant limitations, and further research is required for further confirmation (Figure 8).

## Conclusion

In conclusion, BA may alleviate CCL4-induced HF by modifying the variety, associated functions, and structures of gut microbiota, as well as HF-related liver lesions by influencing gut-related inflammatory responses. These findings show that BA may exert its therapeutic impact by modulating gut microbiota alterations through the liver-gut axis *via* the PI3K/AkT, IL-17, and VEGF signaling pathways. This work may offer an experimental basis for BA research and implementation in HF, establish a link between intestinal flora and HF, and demonstrate that BA has a beneficial therapeutic effect.

## Data availability statement

The datasets presented in this study can be found in online repositories. The names of the repository/repositories and accession number(s) can be found below: Mendeley Data repository (DOI: 10.17632/cx7kg2n2nh.1) (https://data.mendeley.com/datasets/cx7kg2n2nh/1).

## Ethics statement

The animal study was reviewed and approved by this experiment was approved by the Ethical Committee of Heilongjiang university of Chinese medicine (Approved No. DXLL2020081601).

## Author contributions

SjL, PC, ZL, and SmL conceived the idea and designed the study. SjL and SM wrote the original draft. XJ and JW validated the results. SjL, SM, and SmL reviewed and edited the paper. All authors approved the final manuscript.

## Funding

This study was supported by the Natural Science Foundation of Heilongjiang Province (LH2020H099).

## Conflict of interest

The authors declare that the research was conducted in the absence of any commercial or financial relationships that could be construed as a potential conflict of interest.

## Publisher’s note

All claims expressed in this article are solely those of the authors and do not necessarily represent those of their affiliated organizations, or those of the publisher, the editors and the reviewers. Any product that may be evaluated in this article, or claim that may be made by its manufacturer, is not guaranteed or endorsed by the publisher.

## Supplementary material

The Supplementary material for this article can be found online at: https://www.frontiersin.org/articles/10.3389/fmicb.2022.1051100/full#supplementary-material

## Footnotes

^1^ https://pubchem.nlm.nih.gov/

^2^ http://www.lilab-ecust.cn/pharmmapper/

^3^ http://www.tcmip.cn/ETCM/index.php/Home/Index/

^4^ www.swisstargetprediction.ch/

^5^ https://www.uniprot.org/

^6^ https://omim.org/

^7^ https://www.genecards.org

^8^ https://cn.string-db.org/

^9^ https://www.proteinatlas.org
