# Supplementary material for: Exploration of the potential mechanism of Baicalin for hepatic fibrosis based on network pharmacology, gut microbiota, and experimental validation
Source: Front Microbiol. 2023 Jan 4;13:1051100. doi: 10.3389/fmicb.2022.1051100 (PMC9846333; doi:10.3389/fmicb.2022.1051100)
Supplement: Supplementary file 1 [file Data_Sheet_1.PDF]

id	name
baicalin	CASP8
baicalin	BECN1
baicalin	TNF
baicalin	CASP3
baicalin	CYP2E1
baicalin	PDGFB
baicalin	CDX2
baicalin	REN
baicalin	FOXP3
baicalin	ACTB
baicalin	AHR
baicalin	AKT1
baicalin	CA2
baicalin	CA4
baicalin	CDK6
baicalin	CEBPB
baicalin	CSNK2A1
baicalin	DNMT1
baicalin	ESR1
baicalin	ESR2
baicalin	HSP90AA1
baicalin	JAK1
baicalin	NR1I2
baicalin	PIK3CG
baicalin	PRKACA
baicalin	PRKCA
baicalin	PRKCB
baicalin	SHBG
baicalin	AKR1B1
baicalin	ADORA1
baicalin	IL2
baicalin	XDH
baicalin	EGFR
baicalin	ACHE
baicalin	NOX4
baicalin	ALDH2
baicalin	FDFT1
baicalin	P2RY2
baicalin	MAPK14
baicalin	HK2
baicalin	PTGS2
baicalin	POLG
baicalin	FGF2

baicalin	GAA
baicalin	ALOX5
baicalin	POLD1
baicalin	TYMS
baicalin	SELP
baicalin	NPC1L1
baicalin	ADCY10
baicalin	ABCB1
baicalin	F10
baicalin	DHFR
baicalin	ANPEP
baicalin	PTPN22
baicalin	ITGB1
baicalin	CMA1
baicalin	IDO1
baicalin	CASP1
baicalin	EZH2
baicalin	EHMT2
baicalin	NR1H2
baicalin	BCHE
baicalin	PPIA
baicalin	FAP
baicalin	TTR
baicalin	PGR
baicalin	TGFBR2
baicalin	LCN2
baicalin	GSTP1
baicalin	GBA
baicalin	CTSD
baicalin	CCNA2
baicalin	CDK2
baicalin	HSPA8
baicalin	PLAU
baicalin	PAH
baicalin	SRC
baicalin	BMP7
baicalin	MAOB
baicalin	PRDX5
baicalin	APCS
baicalin	PYGL
baicalin	MMP13
baicalin	MMP8
baicalin	FABP4
baicalin	ALB

baicalin APOA2
baicalin PPARG
baicalin CYP19A1
baicalin GSK3B
baicalin PLA2G2A
baicalin SULT2A1
baicalin AR
baicalin KDR
baicalin OTC
baicalin F2
baicalin TPI1
baicalin ADAM17
baicalin NR3C2
baicalin NOS3
baicalin GSR
baicalin LTF
baicalin SOD2
baicalin TYMP
baicalin AHCY
baicalin CTSB
baicalin DPP4
baicalin MIF
baicalin MMP3
baicalin PDE5A
baicalin CRAT
baicalin GALK1
baicalin FGFR1
baicalin IGF1R
baicalin MAPK8
baicalin CHIT1
baicalin GATM
baicalin MTHFD1
baicalin SERPINA1
baicalin ANXA5
baicalin ISG20
baicalin NQO1
baicalin CYP2C9
baicalin PCK1
baicalin PPARD
baicalin GPI
baicalin PLG
baicalin MET
baicalin MAPK10
baicalin HMGCR

baicalin	RNASE3
baicalin	MMP12
baicalin	YARS1
baicalin	HADH
baicalin	HSD11B1
baicalin	MAN1B1
baicalin	PLK1
baicalin	ARSA
baicalin	RAB5A
baicalin	RAB11A
baicalin	IGF1
baicalin	MMP7
baicalin	RHOA
baicalin	NOS2
baicalin	RAC2
baicalin	SYK
baicalin	GC
baicalin	F7
baicalin	AGXT
baicalin	FHIT
baicalin	XIAP
baicalin	ADK
baicalin	ITK
baicalin	CCL5
baicalin	EPHX2
baicalin	LYZ
baicalin	HRAS
baicalin	CTNNA1
baicalin	GALE
baicalin	FGFR2
baicalin	FGF1
baicalin	TGM2
baicalin	PTK2
baicalin	CBS
baicalin	GCK
baicalin	MDM2
baicalin	ADH1C
baicalin	HEXB
baicalin	RXRA
baicalin	PTPN11
baicalin	C1S
baicalin	PARP1
baicalin	RBP4
baicalin	ELANE

baicalin	GSTA1
baicalin	JAK2
baicalin	JAK3
baicalin	KIT
baicalin	ALDOA
baicalin	PPARA
baicalin	MMP9
baicalin	EPHA2
baicalin	LGALS3
baicalin	NR1H3
baicalin	AKR1C2
baicalin	MME
baicalin	FECH
baicalin	TGFBR1
baicalin	CAT
baicalin	INSR
CASP8	hepatic fibrosis
BECN1	hepatic fibrosis
TNF	hepatic fibrosis
CASP3	hepatic fibrosis
CYP2E1	hepatic fibrosis
PDGFB	hepatic fibrosis
CDX2	hepatic fibrosis
REN	hepatic fibrosis
FOXP3	hepatic fibrosis
ACTB	hepatic fibrosis
AHR	hepatic fibrosis
AKT1	hepatic fibrosis
CA2	hepatic fibrosis
CA4	hepatic fibrosis
CDK6	hepatic fibrosis
CEBPB	hepatic fibrosis
CSNK2A1	hepatic fibrosis
DNMT1	hepatic fibrosis
ESR1	hepatic fibrosis
ESR2	hepatic fibrosis
HSP90AA1	hepatic fibrosis
JAK1	hepatic fibrosis
NR1I2	hepatic fibrosis
PIK3CG	hepatic fibrosis
PRKACA	hepatic fibrosis
PRKCA	hepatic fibrosis
PRKCB	hepatic fibrosis
SHBG	hepatic fibrosis

AKR1B1	hepatic fibrosis
ADORA1	hepatic fibrosis
IL2	hepatic fibrosis
XDH	hepatic fibrosis
EGFR	hepatic fibrosis
ACHE	hepatic fibrosis
NOX4	hepatic fibrosis
ALDH2	hepatic fibrosis
FDFT1	hepatic fibrosis
P2RY2	hepatic fibrosis
MAPK14	hepatic fibrosis
HK2	hepatic fibrosis
PTGS2	hepatic fibrosis
POLG	hepatic fibrosis
FGF2	hepatic fibrosis
GAA	hepatic fibrosis
ALOX5	hepatic fibrosis
POLD1	hepatic fibrosis
TYMS	hepatic fibrosis
SELP	hepatic fibrosis
NPC1L1	hepatic fibrosis
ADCY10	hepatic fibrosis
ABCB1	hepatic fibrosis
F10	hepatic fibrosis
DHFR	hepatic fibrosis
ANPEP	hepatic fibrosis
PTPN22	hepatic fibrosis
ITGB1	hepatic fibrosis
CMA1	hepatic fibrosis
IDO1	hepatic fibrosis
CASP1	hepatic fibrosis
EZH2	hepatic fibrosis
EHMT2	hepatic fibrosis
NR1H2	hepatic fibrosis
BCHE	hepatic fibrosis
PPIA	hepatic fibrosis
FAP	hepatic fibrosis
TTR	hepatic fibrosis
PGR	hepatic fibrosis
TGFBR2	hepatic fibrosis
LCN2	hepatic fibrosis
GSTP1	hepatic fibrosis
GBA	hepatic fibrosis
CTSD	hepatic fibrosis

CCNA2	hepatic fibrosis
CDK2	hepatic fibrosis
HSPA8	hepatic fibrosis
PLAU	hepatic fibrosis
PAH	hepatic fibrosis
SRC	hepatic fibrosis
BMP7	hepatic fibrosis
MAOB	hepatic fibrosis
PRDX5	hepatic fibrosis
APCS	hepatic fibrosis
PYGL	hepatic fibrosis
MMP13	hepatic fibrosis
MMP8	hepatic fibrosis
FABP4	hepatic fibrosis
ALB	hepatic fibrosis
APOA2	hepatic fibrosis
PPARG	hepatic fibrosis
CYP19A1	hepatic fibrosis
GSK3B	hepatic fibrosis
PLA2G2A	hepatic fibrosis
SULT2A1	hepatic fibrosis
AR	hepatic fibrosis
KDR	hepatic fibrosis
OTC	hepatic fibrosis
F2	hepatic fibrosis
TPI1	hepatic fibrosis
ADAM17	hepatic fibrosis
NR3C2	hepatic fibrosis
NOS3	hepatic fibrosis
GSR	hepatic fibrosis
LTF	hepatic fibrosis
SOD2	hepatic fibrosis
TYMP	hepatic fibrosis
AHCY	hepatic fibrosis
CTSB	hepatic fibrosis
DPP4	hepatic fibrosis
MIF	hepatic fibrosis
MMP3	hepatic fibrosis
PDE5A	hepatic fibrosis
CRAT	hepatic fibrosis
GALK1	hepatic fibrosis
FGFR1	hepatic fibrosis
IGF1R	hepatic fibrosis
MAPK8	hepatic fibrosis

CHIT1	hepatic fibrosis
GATM	hepatic fibrosis
MTHFD1	hepatic fibrosis
SERPINA1	hepatic fibrosis
ANXA5	hepatic fibrosis
ISG20	hepatic fibrosis
NQO1	hepatic fibrosis
CYP2C9	hepatic fibrosis
PCK1	hepatic fibrosis
PPARD	hepatic fibrosis
GPI	hepatic fibrosis
PLG	hepatic fibrosis
MET	hepatic fibrosis
MAPK10	hepatic fibrosis
HMGCR	hepatic fibrosis
RNASE3	hepatic fibrosis
MMP12	hepatic fibrosis
YARS1	hepatic fibrosis
HADH	hepatic fibrosis
HSD11B1	hepatic fibrosis
MAN1B1	hepatic fibrosis
PLK1	hepatic fibrosis
ARSA	hepatic fibrosis
RAB5A	hepatic fibrosis
RAB11A	hepatic fibrosis
IGF1	hepatic fibrosis
MMP7	hepatic fibrosis
RHOA	hepatic fibrosis
NOS2	hepatic fibrosis
RAC2	hepatic fibrosis
SYK	hepatic fibrosis
GC	hepatic fibrosis
F7	hepatic fibrosis
AGXT	hepatic fibrosis
FHIT	hepatic fibrosis
XIAP	hepatic fibrosis
ADK	hepatic fibrosis
ITK	hepatic fibrosis
CCL5	hepatic fibrosis
EPHX2	hepatic fibrosis
LYZ	hepatic fibrosis
HRAS	hepatic fibrosis
CTNNA1	hepatic fibrosis
GALE	hepatic fibrosis

FGFR2	hepatic fibrosis
FGF1	hepatic fibrosis
TGM2	hepatic fibrosis
PTK2	hepatic fibrosis
CBS	hepatic fibrosis
GCK	hepatic fibrosis
MDM2	hepatic fibrosis
ADH1C	hepatic fibrosis
HEXB	hepatic fibrosis
RXRA	hepatic fibrosis
PTPN11	hepatic fibrosis
C1S	hepatic fibrosis
PARP1	hepatic fibrosis
RBP4	hepatic fibrosis
ELANE	hepatic fibrosis
GSTA1	hepatic fibrosis
JAK2	hepatic fibrosis
JAK3	hepatic fibrosis
KIT	hepatic fibrosis
ALDOA	hepatic fibrosis
PPARA	hepatic fibrosis
MMP9	hepatic fibrosis
EPHA2	hepatic fibrosis
LGALS3	hepatic fibrosis
NR1H3	hepatic fibrosis
AKR1C2	hepatic fibrosis
MME	hepatic fibrosis
FECH	hepatic fibrosis
TGFBR1	hepatic fibrosis
CAT	hepatic fibrosis
INSR	hepatic fibrosis

RELA	RELA	64
STAT1	STAT1	60
HSP90AA1	HSP90AA1	85
SOD1	SOD1	35
JUN	JUN	101
MPO	MPO	53
CYP2E1	CYP2E1	31
CASP3	CASP3	104
MAPK8	MAPK8	65
PPARG	PPARG	99
CYCS	CYCS	73

TNF	TNF	116
IL1B	IL1B	106
HMOX1	HMOX1	76
PTGS2	PTGS2	94
MYC	MYC	92
CAT	CAT	83
UGT1A1	UGT1A1	
TP53	TP53	110
PTGS1	PTGS1	
PPARA	PPARA	76
MMP2	MMP2	75
MGAM	MGAM	13
CTNNB1	CTNNB1	93
HMGCR	HMGCR	21
MTTP	MTTP	9
OLR1	OLR1	22
CTSD	CTSD	25
F2	F2	36
HSPA5	HSPA5	52
CYP1A1	CYP1A1	27
ESR1	ESR1	92
OPRM1	OPRM1	6
HSD11B2	HSD11B2	
MT-ND6	MT-ND6	3
NR3C2	NR3C2	
F7	F7	11
SOAT1	SOAT1	4
CDKN1A	CDKN1A	54
ALOX5	ALOX5	23
F3	F3	34
NOS2	NOS2	
CXCL10	CXCL10	43
NCF1	NCF1	36
ODC1	ODC1	11
MAPK14	MAPK14	64
NFKBIA	NFKBIA	73
XDH	XDH	
AKR1B1	AKR1B1	
NFE2L2	NFE2L2	54
NQO1	NQO1	34
CYP3A4	CYP3A4	28
CYP2C9	CYP2C9	17
GSTM1	GSTM1	18
AHR	AHR	39

EGFR	EGFR	95
IL10	IL10	79
HIF1A	HIF1A	91
IKBKB	IKBKB	56
IGF2	IGF2	39
CYP19A1	CYP19A1	
JAK2	JAK2	54
MMP9	MMP9	92
PTPN1	PTPN1	40
DPP4	DPP4	
SELE	SELE	47
CXCL8	CXCL8	83
NOS3	NOS3	83
VCAM1	VCAM1	64
ICAM1	ICAM1	70
STAT3	STAT3	91
IL1A	IL1A	47
MAPK3	MAPK3	98
CRP	CRP	57
IL4	IL4	62
IFNG	IFNG	63
IL2	IL2	61
CCL2	CCL2	80
SERPINE1	SERPINE1	64
TGFB1	TGFB1	59
IL6	IL6	117
FN1	FN1	91
CAV1	CAV1	62
PON1	PON1	21
GSR	GSR	
LDLR	LDLR	32
AKT1	AKT1	123
ADIPOQ	ADIPOQ	63
SREBF1	SREBF1	44
SLC2A4	SLC2A4	44
FASN	FASN	32
APOB	APOB	
ACACA	ACACA	15

**pathway**	**Fold Enrichment**	**PValue**	**Count**
Pathways in cancer	4.363551	2.81E-19	50
Lipid and atherosclerosis	5.604017	3.33E-12	26

Proteoglycans in cancer	5.65133	8.13E-12	25
PI3K-Akt signaling pathway	4.189009	1.33E-11	32
EGFR tyrosine kinase inhibitor resistance	9.385501	8.42E-11	16
Ras signaling pathway	4.929884	1.50E-10	25
Prostate cancer	8.121603	1.78E-10	17
Rap1 signaling pathway	5.075433	5.73E-10	23
MAPK signaling pathway	4.255798	5.86E-10	27
Fluid shear stress and atherosclerosis	6.000981	5.61E-09	18
Hepatitis B	5.435045	9.21E-09	19
FoxO signaling pathway	6.013706	1.65E-08	17
Endocrine resistance	7.092996	1.77E-08	15
Adherens junction	8.484955	2.78E-08	13
C-type lectin receptor signaling pathway	6.683785	3.86E-08	15
Focal adhesion	4.611036	5.03E-08	20
IL-17 signaling pathway	6.901838	8.68E-08	14
Toxoplasmosis	6.206372	1.00E-07	15
Chemical carcinogenesis - receptor activation	4.371784	1.18E-07	20
AGE-RAGE signaling pathway in diabetic complications	6.487727	1.83E-07	14
Human cytomegalovirus infection	4.119192	3.02E-07	20
Diabetic cardiomyopathy	4.337326	3.05E-07	19
Melanoma	7.723485	3.15E-07	12
Metabolic pathways	1.866885	3.21E-07	62
VEGF signaling pathway	8.639831	4.05E-07	11
Insulin resistance	6.007155	4.57E-07	14
Relaxin signaling pathway	5.388478	5.94E-07	15
Kaposi sarcoma-associated herpesvirus infection	4.299672	7.84E-07	18
Chemical carcinogenesis - reactive oxygen species	3.948329	1.23E-06	19
Prolactin signaling pathway	7.282143	2.09E-06	11
Th17 cell differentiation	5.578072	3.04E-06	13

GO

**Class**	**Description**	**Enrichment**	**pvalue**	**count**
BP	positive regulation of phosphatidylinositol 3-kinase signaling	21.97894737	3.93E-18	18
BP	positive regulation of cell migration	9.501315789	8.45E-16	24
BP	positive regulation of MAP kinase activity	20.26947368	1.69E-15	16
BP	positive regulation of smooth muscle cell proliferation	21.71729323	5.69E-15	15
BP	negative regulation of apoptotic process	5.972943766	6.36E-15	31
BP	response to ethanol	13.7161852	1.60E-14	18
BP	positive regulation of cell proliferation	5.722711149	1.96E-14	31
BP	positive regulation of protein kinase B signaling	13.46019737	1.40E-13	17

BP	response to drug	7.609700018	1.31E-12	22
BP	proteolysis	6.194826921	2.22E-12	25
CC	extracellular exosome	3.23772655	9.32E-19	67
CC	extracellular space	3.43252131	2.05E-18	62
CC	extracellular region	3.225071814	9.88E-18	64
CC	cytosol	2.015679084	2.83E-15	103
CC	ficolin-1-rich granule lumen	12.96571525	9.69E-12	15
CC	focal adhesion	5.561394843	4.80E-10	22
CC	receptor complex	7.902912153	1.90E-09	16
CC	mitochondrion	2.908939873	3.59E-09	39
CC	cytoplasm	1.712622783	1.22E-08	89
CC	membrane raft	6.405373271	9.98E-08	15
MF	identical protein binding	3.279061873	8.31E-16	57
MF	protein tyrosine kinase activity	13.94504934	5.08E-13	16
MF	transmembrane receptor protein tyrosine kinase activity	13.58440152	7.52E-13	16
MF	enzyme binding	6.060732984	1.06E-11	24
MF	endopeptidase activity	14.71643498	7.29E-11	13
MF	serine-type endopeptidase activity	8.630296324	1.32E-10	17
MF	ATP binding	2.806621816	7.07E-10	44
MF	protein homodimerization activity	4.005778989	7.38E-10	29
MF	protein kinase activity	5.197198469	1.07E-08	20
MF	protease binding	10.84259571	1.36E-08	12
