# Supplementary material for: Exploration of the potential mechanism of Baicalin for hepatic fibrosis based on network pharmacology, gut microbiota, and experimental validation
Source: Front Microbiol. 2023 Jan 4;13:1051100. doi: 10.3389/fmicb.2022.1051100 (PMC9846333; doi:10.3389/fmicb.2022.1051100)
Supplement: Supplementary file 2 [file Data_Sheet_2.PDF]

Liver (HE)

| | Con | M | B |
|---|---|---|---|
| 1 | 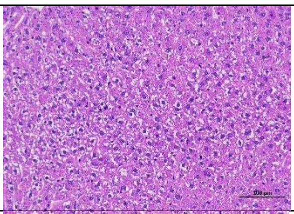 | 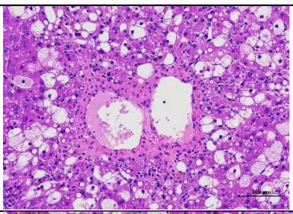 | 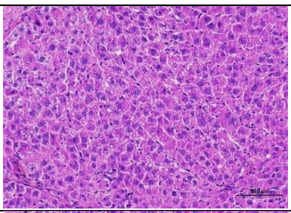 |
| 2 | 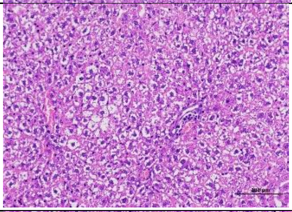 | 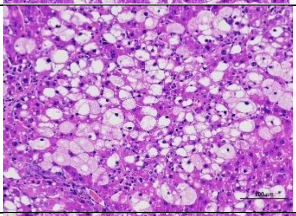 | 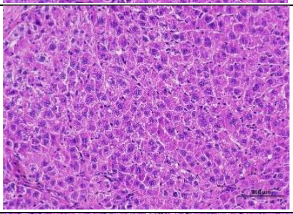 |
| 3 | 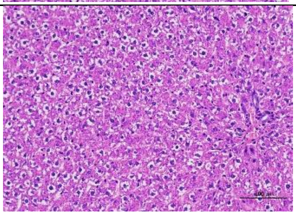 | 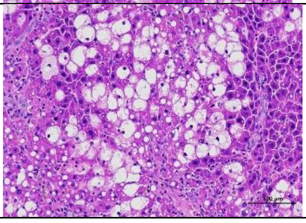 | 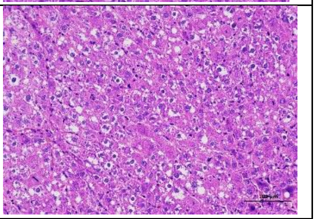 |

Liver(Masson)

| | Con | M | B |
|---|---|---|---|
| 1 | 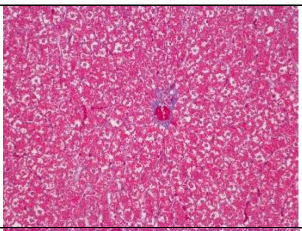 | 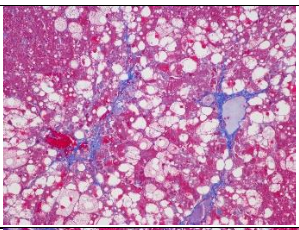 | 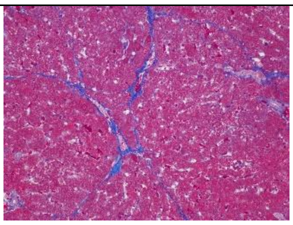 |
| 2 | 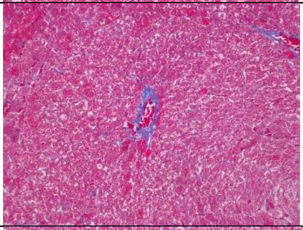 | 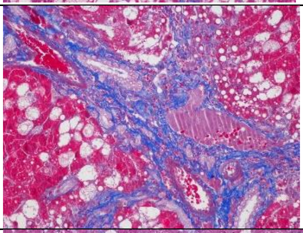 | 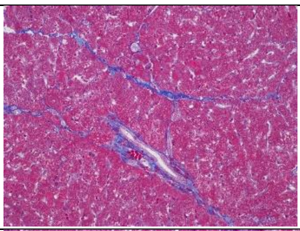 |
| 3 | 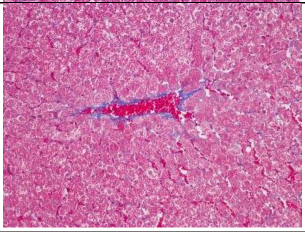 | 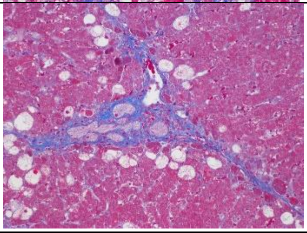 | 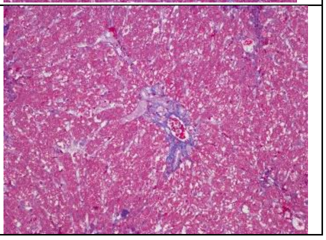 |

intestinal wall(HE)

| | Con | M | B |
|---|---|---|---|
| 1 | 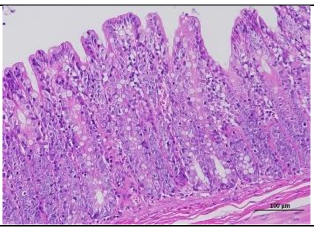 | 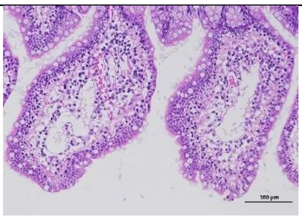 | 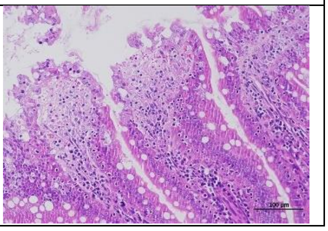 |

| | | | |
|---|---|---|---|
| 2 | 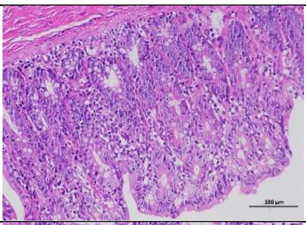 | 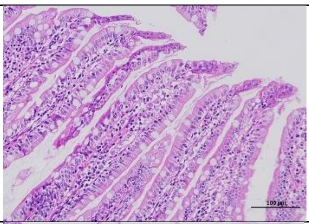 | 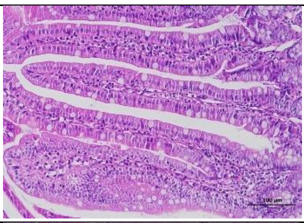 |
| 3 | 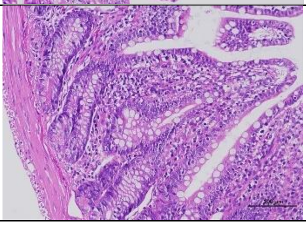 | 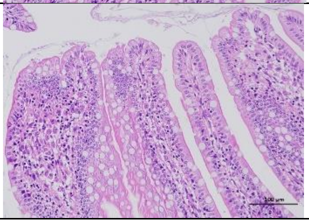 | 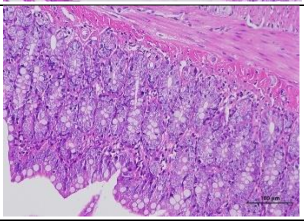 |

The Human Protein Altas

| | MGAM | PTGS1 | MTTP | HSD11B2 | APOB | NOS2 | XDH | DPP4 | UGT1A1 |
|---|---|---|---|---|---|---|---|---|---|
| Normal | 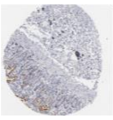 | 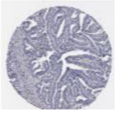 | 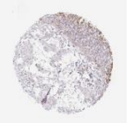 | 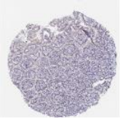 | 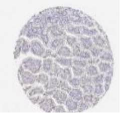 | 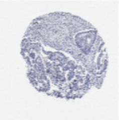 | 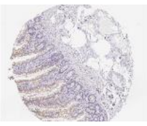 | 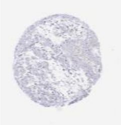 | 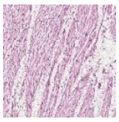 |
| Patholog | 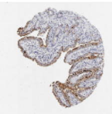 | 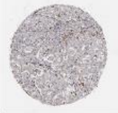 | 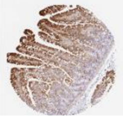 | 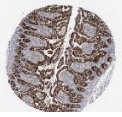 | 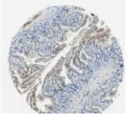 | 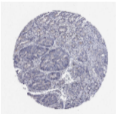 | 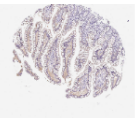 | 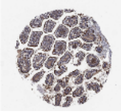 | 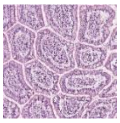 |

ZO-1

| | Con | M | B |
|---|---|---|---|
| 1 | 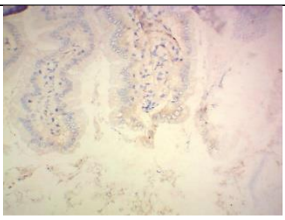 | 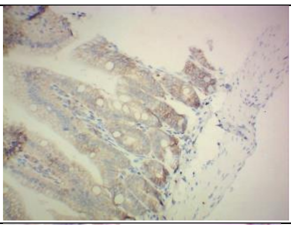 | 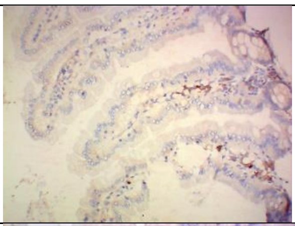 |
| 2 | 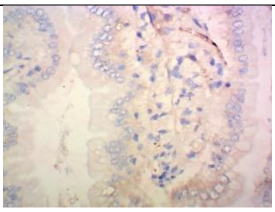 | 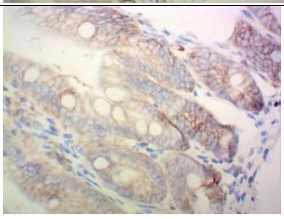 | 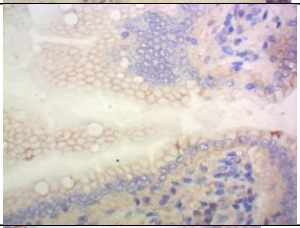 |
| 3 | 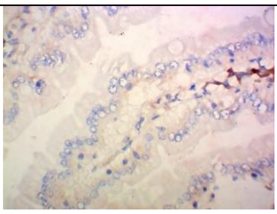 | 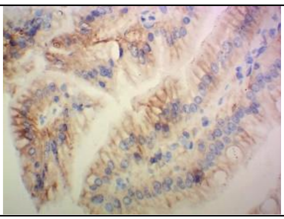 | 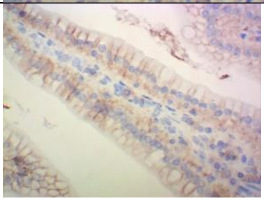 |

Claudin-1

| | Con | M | B |
|---|---|---|---|
| 1 | 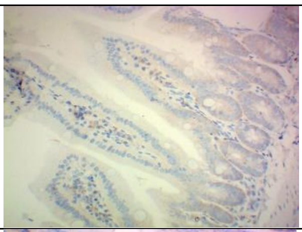 | 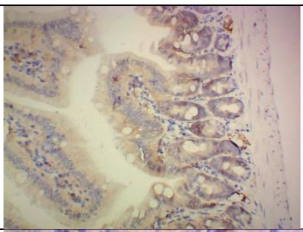 | 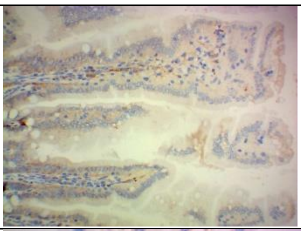 |
| 2 | 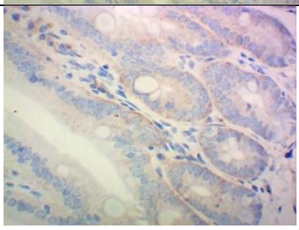 | 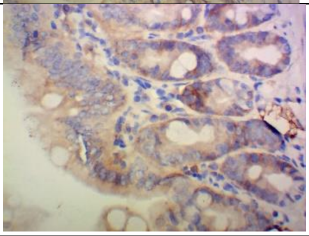 | 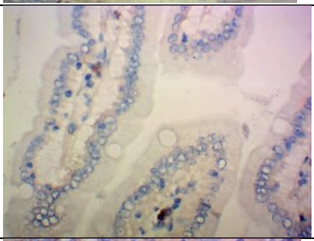 |
| 3 | 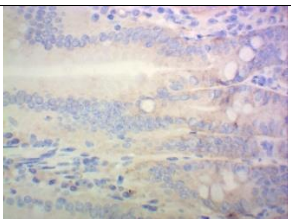 | 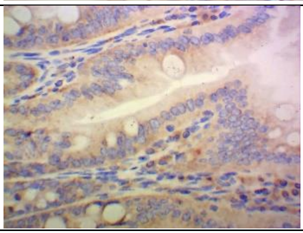 | 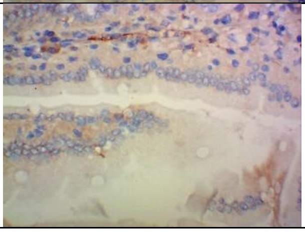 |

Western Blot

| | | | |
|---|---|---|---|
| p-AKT | 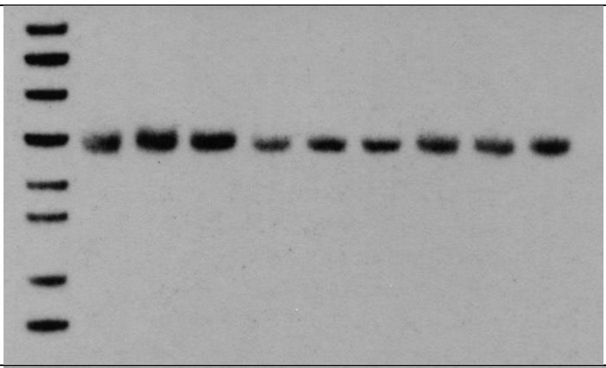 | p-PI3K | 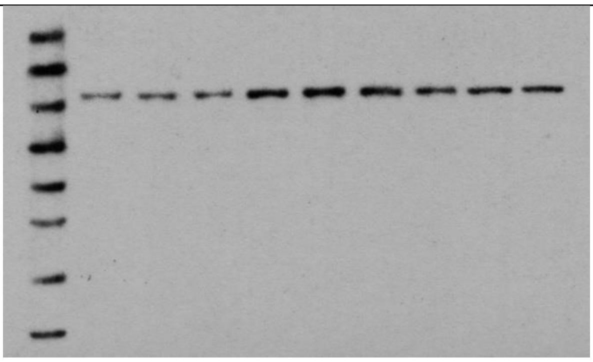 |
| p-mTOR | 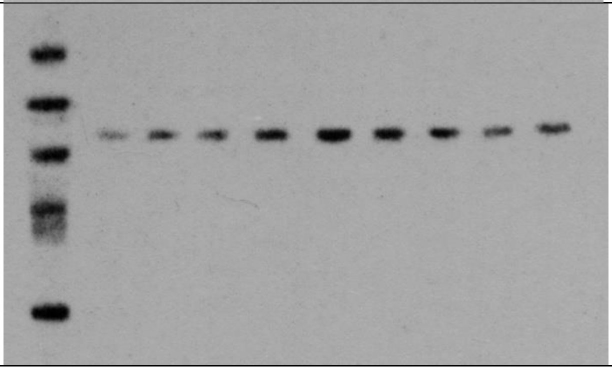 | VEGF | 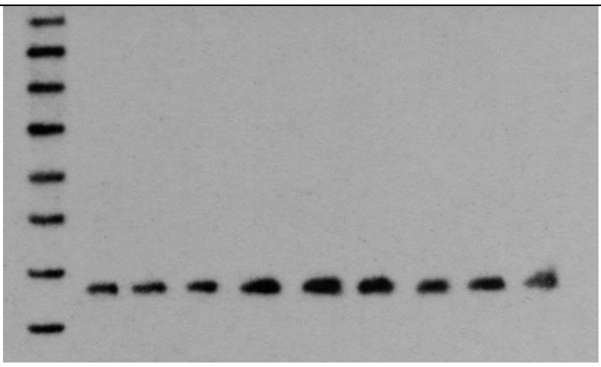 |

IL-17

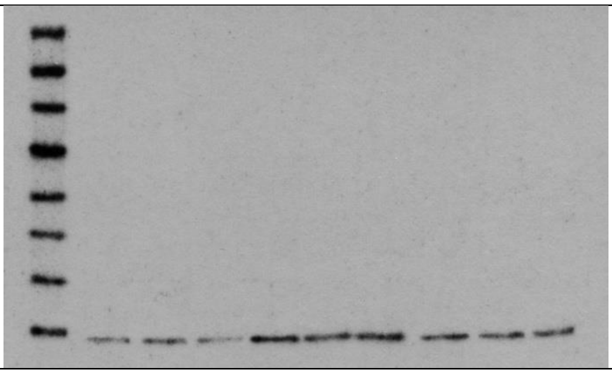

β-actin

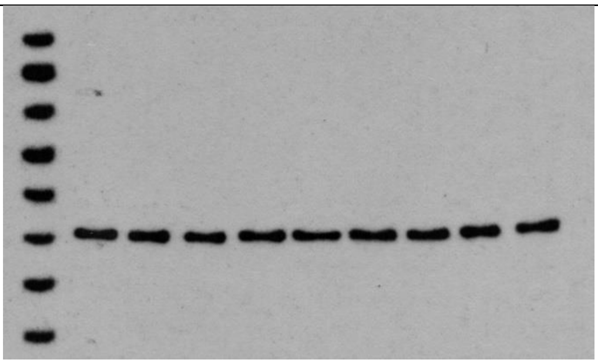

| | K：空白组 | | | M：模型组 | | | H：给药组 | | |
|---|---|---|---|---|---|---|---|---|---|
| p-AKT | 28.25 | 35.18 | 61.43 | 121.82 | 140.22 | 130.02 | 75.27 | 90.72 | 110.03 |
| p-PI3K | 38.89 | 47.80 | 47.51 | 157.32 | 192.49 | 143.21 | 83.23 | 97.94 | 87.89 |
| p-mTOR | 35.09 | 49.47 | 50.97 | 105.62 | 177.60 | 115.84 | 92.34 | 50.51 | 61.59 |
| VEGF | 107.06 | 114.32 | 134.23 | 294.61 | 286.22 | 244.84 | 137.95 | 162.96 | 125.41 |
| IL-17 | 89.34 | 235.39 | 174.03 | 873.16 | 433.29 | 614.48 | 209.27 | 339.60 | 409.78 |
| β-actin | 229.61 | 305.42 | 271.57 | 336.46 | 307.37 | 317.01 | 274.19 | 292.60 | 312.27 |

**phylum level**

| ID | Con1 | Con2 | Con3 | Con4 | Con5 | M1 | M2 | M3 | M4 | M5 | B1 | B2 | B3 | B4 | B5 |
|---|---|---|---|---|---|---|---|---|---|---|---|---|---|---|---|
| Firmicutes | 0.73784736 | 0.97828177 | 0.65128415 | 0.8475732 | 0.75529341 | 0.53388361 | 0.75554986 | 0.679629 | 0.72379197 | 0.68161238 | 0.88601289 | 0.96448376 | 0.97193629 | 0.96738409 | 0.9595677 |
| Bacteroidetes | 0.20568542 | 0.00515601 | 0.31920598 | 0.10611713 | 0.19816783 | 0.4387224 | 0.2258154 | 0.2541005 | 0.26231321 | 0.30094324 | 0.054874 | 0.01069977 | 0.01371973 | 0.01587026 | 0.02075986 |
| Proteobacteria | 0.00328973 | 0.00072947 | 0.00419523 | 0.02071801 | 0.01296344 | 0.02023646 | 0.00794057 | 0.03979041 | 0.00483214 | 0.00204657 | 0.00933776 | 0.00767338 | 0.00416076 | 0.00330177 | 0.00557989 |
| Actinobacteria | 0.02935599 | 0.00815677 | 0.0138545 | 0.01534296 | 0.01421658 | 0.00271149 | 0.00371414 | 0.01712473 | 0.00304677 | 0.00546276 | 0.01041219 | 0.00861059 | 0.00360924 | 0.00703505 | 0.00653209 |
| Tenericutes | 0.01093351 | 0.00251998 | 0.00587332 | 0.00387084 | 0.01039236 | 0.00223299 | 0.00275359 | 0.00072273 | 0.00065981 | 0.00237717 | 0.00478609 | 0.00515464 | 0.00493509 | 0.00539131 | 0.00658922 |
| Verrucomicrobia | 0 | 0 | 0.00014325 | 0.00004011 | 0.00019445 | 0 | 0 | 0.00176668 | 0.00042694 | 0.00281797 | 0.02732956 | 0.00144486 | 0.00092073 | 0.00031149 | 0.00011426 |
| Cyanobacteria | 0.00536032 | 0.00185683 | 0.00102323 | 0.0011432 | 0.00120992 | 0.00007975 | 0.00224129 | 0.00240911 | 0.00007762 | 0.00042506 | 0.00050791 | 0.00089816 | 0 | 0.00022842 | 0.00028566 |
| Deferribacteres | 0 | 0 | 0.00004093 | 0.00006017 | 0 | 0 | 0 | 0 | 0 | 0 | 0.00507912 | 0.00009763 | 0 | 0 | 0 |
| TM7 | 0.00030962 | 0.00127657 | 0.00036836 | 0.00036101 | 0 | 0 | 0 | 0 | 0 | 0 | 0.0003321 | 0.00025383 | 0 | 0.00014536 | 0.00007618 |
| Spirochaetes | 0 | 0 | 0 | 0 | 0 | 0.00051837 | 0.00027749 | 0.00094357 | 0.00065981 | 0.00007871 | 0 | 0 | 0 | 0 | 0 |
| Others | 0.00721805 | 0.00202261 | 0.00401105 | 0.00477337 | 0.00756201 | 0.00161493 | 0.00170765 | 0.00351328 | 0.00419173 | 0.00423614 | 0.00132838 | 0.00068338 | 0.00071817 | 0.00033225 | 0.00049514 |

**genus level**

| ID | Con1 | Con2 | Con3 | Con4 | Con5 | M1 | M2 | M3 | M4 | M5 | B1 | B2 | B3 | B4 | B5 |
|---|---|---|---|---|---|---|---|---|---|---|---|---|---|---|---|
| Lactobacillus | 0.4758766 | 0.5535993 | 0.2543334 | 0.4679503 | 0.3288782 | 0.08621768 | 0.03750427 | 0.1196322 | 0.06109451 | 0.02108279 | 0.2993163 | 0.3180217 | 0.219133 | 0.4912534 | 0.2811274 |
| Oscillospira | 0.01269448 | 0.12346497 | 0.04996623 | 0.03671881 | 0.00423818 | 0.2420785 | 0.1994845 | 0.2876899 | 0.3231207 | 0.1718149 | 0.11122196 | 0.14062158 | 0.14111542 | 0.09951485 | 0.08291494 |

| | | | | | | | | | | | | | | | |
|---|---|---|---|---|---|---|---|---|---|---|---|---|---|---|---|
| Bacteroides | 0.00771635 | 0.00201109 | 0.00178621 | 0.00132901 | 0.0038088 | 0.34425903 | 0.12254525 | 0.11132079 | 0.07273433 | 0.11157428 | 0.00963697 | 0.00056368 | 0.00771513 | 0.00409146 | 0.01300665 |
| Ruminococcaceae_Ruminococcus | 0.0511843 | 0.00981465 | 0.03845288 | 0.02567188 | 0.04621467 | 0.00382798 | 0.01910434 | 0.01527775 | 0.030817 | 0.02010359 | 0.04586833 | 0.02893627 | 0.02123193 | 0.01640502 | 0.02683298 |
| Clostridiaceae_Clostridium | 0.06937456 | 0.03670546 | 0.02238821 | 0.02344565 | 0.02573243 | 0.00496441 | 0.00574197 | 0.01170424 | 0.00279449 | 0.01621511 | 0.01826529 | 0.0163816 | 0.00173096 | 0.00618822 | 0.01426395 |
| [Ruminococcus] | 0.00563124 | 0.00270234 | 0.00638494 | 0.00282792 | 0.00862069 | 0.05745958 | 0.03146346 | 0.01419365 | 0.00972249 | 0.00266054 | 0.01090057 | 0.0128085 | 0.00279901 | 0.0076003 | 0.00704628 |
| Coprococcus | 0.0049733 | 0.00591863 | 0.00321293 | 0.00523466 | 0.0075404 | 0.00346911 | 0.01163337 | 0.01039931 | 0.01618475 | 0.00908361 | 0.01668295 | 0.01329663 | 0.00966762 | 0.01146275 | 0.01437821 |
| Dorea | 0.00125784 | 0.00024868 | 0.00096183 | 0.00020056 | 0.00177167 | 0.01832247 | 0.02177254 | 0.01106181 | 0.00405589 | 0.00086586 | 0.01218988 | 0.01269135 | 0.0012706 | 0.00639588 | 0.00580842 |
| rc4-4 | 0.00023222 | 0.00049736 | 0.00049115 | 0.00022062 | 0.00023766 | 0.00045856 | 0.00040557 | 0.00042159 | 0.00120318 | 0.00037783 | 0.00605587 | 0.01727976 | 0.01629684 | 0.0335576 | 0.01279756 |
| Parabacteroides | 0.00112238 | 0 | 0.00212831 | 0.0005014 | 0.00265751 | 0.04707219 | 0.00352203 | 0.00971673 | 0.00599651 | 0.00007871 | 0.00080094 | 0.00005858 | 0.0001289 | 0.00016613 | 0.00005713 |
| Roseburia | 0.0013933 | 0.00051394 | 0.00014325 | 0.00078219 | 0.00079941 | 0.00576191 | 0.01938183 | 0.00044167 | 0.00052397 | 0 | 0.01486618 | 0.01333568 | 0.0046957 | 0.00253343 | 0.00759855 |
| Butyricimonas | 0.00183838 | 0.00049736 | 0.00155531 | 0.0003209 | 0.00274393 | 0.00364854 | 0.02072661 | 0.01411335 | 0.01373957 | 0.00015743 | 0.00085954 | 0.00089816 | 0 | 0.00120442 | 0.00083794 |
| Phascolarctobacterium | 0.00178032 | 0.00117709 | 0.00159623 | 0.00132371 | 0.01220724 | 0.00460554 | 0.00604081 | 0.00295116 | 0.00426936 | 0.00484879 | 0.00310608 | 0.0033388 | 0.00585581 | 0.00622975 | 0.00338983 |
| Adlercreutzia | 0.02068659 | 0.00487417 | 0.00853372 | 0.00902527 | 0.00572552 | 0.000957 | 0.00081113 | 0.00078296 | 0.00034931 | 0.00048803 | 0.00132838 | 0.00343643 | 0.00077341 | 0.00045685 | 0.00097124 |
| CF231 | 0.00476043 | 0.00028184 | 0.00888161 | 0.00395106 | 0.00831821 | 0.00263174 | 0.01013917 | 0.01929293 | 0 | 0 | 0 | 0 | 0 | 0 | 0 |
| Shigella | 0.00141265 | 0.00013263 | 0.0009823 | 0.00040112 | 0.00159882 | 0.0070379 | 0.0007471 | 0.02700207 | 0.00071803 | 0.00073991 | 0.00013675 | 0.0003905 | 0.00011049 | 0 | 0.0003047 |
| Blautia | 0.00094822 | 0.00049736 | 0.00051161 | 0.00022062 | 0.00522859 | 0.02007696 | 0.00147285 | 0.00329245 | 0.00203765 | 0.00007861 | 0.00009768 | 0.00138629 | 0.00300157 | 0.00020766 | 0.00087602 |
| Others | 0.337117 | 0.257063 | 0.59769 | 0.419874 | 0.533678 | 0.14715046 | 0.487503171 | 0.34070582 | 0.4506383 | 0.63983001 | 0.44866633 | 0.416554 | 0.564474 | 0.312732 | 0.527788 |

| | **HA** | | | **LN** | | | **PCⅢ** | | | **CIV** | | |
|---|---|---|---|---|---|---|---|---|---|---|---|---|
| | Con | M | B | Con | M | B | Con | M | B | Con | M | B |
| 1 | 11.2893 | 59.0232 | 26.3467 | 65.1312 | 155.3842 | 130.3782 | 110.293 | 319.1812 | 243.1931 | 37.3892 | 102.3893 | 68.2389 |
| 2 | 14.1898 | 42.3524 | 31.3733 | 89.7237 | 143.1383 | 132.1381 | 120.1831 | 287.1831 | 183.3831 | 40.3289 | 119.3832 | 89.2389 |
| 3 | 22.4921 | 55.5904 | 28.4386 | 75.3264 | 174.2372 | 97.3831 | 105.1831 | 275.1831 | 155.2381 | 67.8923 | 137.3891 | 98.2389 |
| 4 | 10.8931 | 53.2685 | 28.7962 | 82.8642 | 146.2389 | 141.2389 | 96.2831 | 306.3282 | 196.4892 | 73.2389 | 89.3781 | 101.2389 |
| 5 | 15.1378 | 56.547 | 38.2186 | 60.1224 | 170.2389 | 137.2381 | 159.831 | 295.2831 | 206.3892 | 43.8942 | 87.1381 | 65.2389 |
| 6 | 17.3844 | 57.1933 | 25.2375 | 81.0105 | 164.2381 | 126.2371 | 149.1931 | 255.2389 | 231.3892 | 49.8231 | 96.8312 | 74.2389 |
| 7 | 18.3811 | 47.1093 | 22.1378 | 71.2456 | 189.2389 | 135.2389 | 137.1831 | 299.9371 | 221.3892 | 56.3891 | 126.8131 | 79.2389 |
| 8 | 12.1389 | 49.8218 | 26.877 | 93.2389 | 180.1378 | 158.2371 | 129.1821 | 221.2821 | 256.3892 | 40.3892 | 130.8923 | 82.8922 |

| weight | Con | M | B |
|---|---|---|---|
| 2week | 235.75 | 236.875 | 232.125 |
| 4week | 271.625 | 237.75 | 250.875 |

| | | | |
|---|---|---|---|
| 6week | 318.875 | 257.75 | 279.25 |
| 8week | 367.75 | 283.75 | 310.75 |
| | 408.75 | 310.375 | 366.25 |
